# Synthesis of New Benzothiazole Acylhydrazones as Anticancer Agents

**DOI:** 10.3390/molecules23051054

**Published:** 2018-05-01

**Authors:** Derya Osmaniye, Serkan Levent, Abdullah Burak Karaduman, Sinem Ilgın, Yusuf Özkay, Zafer Asım Kaplancıklı

**Affiliations:** 1 Department of Pharmaceutical Chemistry, Faculty of Pharmacy, Anadolu University, 26470 Eskişehir, Turkey; dosmaniye@anadolu.edu.tr (D.O.); serkanlevent@anadolu.edu.tr (S.L.); zakaplan@anadolu.edu.tr (Z.A.K.); 2 Doping and Narcotic Compounds Analysis Laboratory, Faculty of Pharmacy, Anadolu University, 26470 Eskişehir, Turkey; 3 Department of Pharmaceutical Toxicology, Faculty of Pharmacy, Anadolu Universty, 26470 Eskişehir, Turkey; abkaraduman@anadolu.edu.tr (A.B.K.); silgin@anadolu.edu.tr (S.I.)

**Keywords:** benzothiazole, hydrazone, anticancer, C6, A549, MCF-7, HT-29, apoptosis

## Abstract

During the last five decades, a large number of BT (Benzothiazole) derivatives formed one of the eligible structures in medicinal chemistry as anticancer agents. Most of the studies reveal that various substitutions at specific positions on BT scaffold modulate the antitumor property. The potential of BTs encouraged us to synthesize a number of new 2-((5-substitutedbenzothiazol-2-yl)thio)-*N*’-(2-(4-(substitutedphenyl)ethylidene)acetohydrazide derivatives and investigate their probable anticancer activity. 4-Substitued benzaldehyde derivatives (**1a**–**1e**) were afforded by the reaction of appropriate secondary amine and 4-fluorobenzaldehyde in DMF. Equimolar quantitates of 5-substitutedbenzothiazole-2-thiol, ethyl chloroacetate and K_2_CO_3_ were refluxed in acetone to obtain 2-((5-substitutedbenzothiazol-2-yl)thio)acetate derivatives (**2a**,**2b**), which reacted with excess of hydrazine hydrate to get 2-((5-substitutebenzothiazol-2-yl)thio)acetohydrazides (**3a**,**3b**). In the last step, 2-((5-substitutedbenzothiazol-2-yl)thio)-*N*’-(4-substitutedbenzylidene)acetohydrazide derivatives (**4a**–**4j**) were synthesized by the reaction of **1a**–**1e** and **3a**–**3b** in EtOH. The anticancer activity of target compounds was evaluated in three steps. First, an MTT test (3-(4,5-dimethylthiazol-2-yl)-2,5-diphenyltetrazolium bromide) was performed to observe cytotoxic activity of the compounds against carcinogenic C6 (Rat brain glioma cell line), A549 (Human lung adenocarcinoma epithelial cell line), MCF-7 (Human breast adenocarcinoma cell line), and HT-29 (Human colorectal adenocarcinoma cell line) cancer cell lines. Healthy NIH3T3 (Mouse embryo fibroblast cell line) cells were also subjected to MTT assay to determine selectivity of the compounds towards carcinogenic cell lines. Secondly, inhibitory effects of selected compounds **4d**, **4e**, and **4h** on DNA synthesis of C6 cells were investigated. Finally, flow cytometric analysis were performed to identify the death pathway of the carcinogenic cells.

## 1. Introduction

Cancer is still one of the most common reasons of death worldwide. In spite of the widespread research and fast changes in the cancer therapy, there is still a rising need for new treatments [1]. Discovery of new chemotherapeutics are of principal importance due to the essential capability of tumour cells to develop resistance to current agents. The progress of multiple drug resistance to antitumor drugs is a major problem in the chemotherapy. Hence, research for the recognition of novel agents for the managing of cancer is at the critical level [2].

Benzothiazole (BT) compounds display the anticancer activity by acting on various biological targets. Some important instances related to these biotargets are replication and mitosis inhibitors [3], inhibitors of thioredoxin signaling system [4], topoisomerase II inhibitor [5], cytochrome P450 (CYP) inhibitors [6,7,8], tyrosine kinase inhibitors [9], heat shock protein 90 (Hsp90) inhibitors [10], epidermal growth factor receptor (EGFR) inhibitors [11], and cathepsin D inhibitors [12].

Rational drug design is a strategy that is applied in the discovery of novel lead drugs. Drug design methodologies can be classified as ligand based and structure based. The original hit ring system, BT, was extensively modified through a structure-based drug design approach. Previous studies reveal that various groups at specific positions, particularly at the C2 and C6, on BT scaffold modulate the antitumor potency of the scaffold [13]. Most of the reported compounds with an anticancer activity possess a phenyl moiety directly linked to C2 or through a linker group [14]. A literature survey shows that the compounds include amide, urea, and semicarbazone moieties as linkers between BT and phenyl have significant anticancer activity [13,15,16,17].

*N*-acylarylhydrazone scaffold has been widely used as a building block in several antitumor agents owing to its flexible skeleton and the presence of both hydrogen donors and acceptors [15]. Most of the antitumor acylhydrazones have been observed through inducing apoptosis in diverse carcinogenic cells [18,19]. In addition, it has been reported that the compounds include BT and hydrazone moieties on the same structure have significant anticancer activity [20,21,22,23,24,25].

In light of the knowledge above and with a goal to discover a novel class of anticancer agents, we report herein the synthesis and antitumor effect of new compounds, including acylhydrazone moiety as a linker between BT and phenyl scaffolds.

## 2. Results and Discussion

### 2.1. Chemistry

**Scheme 1:**
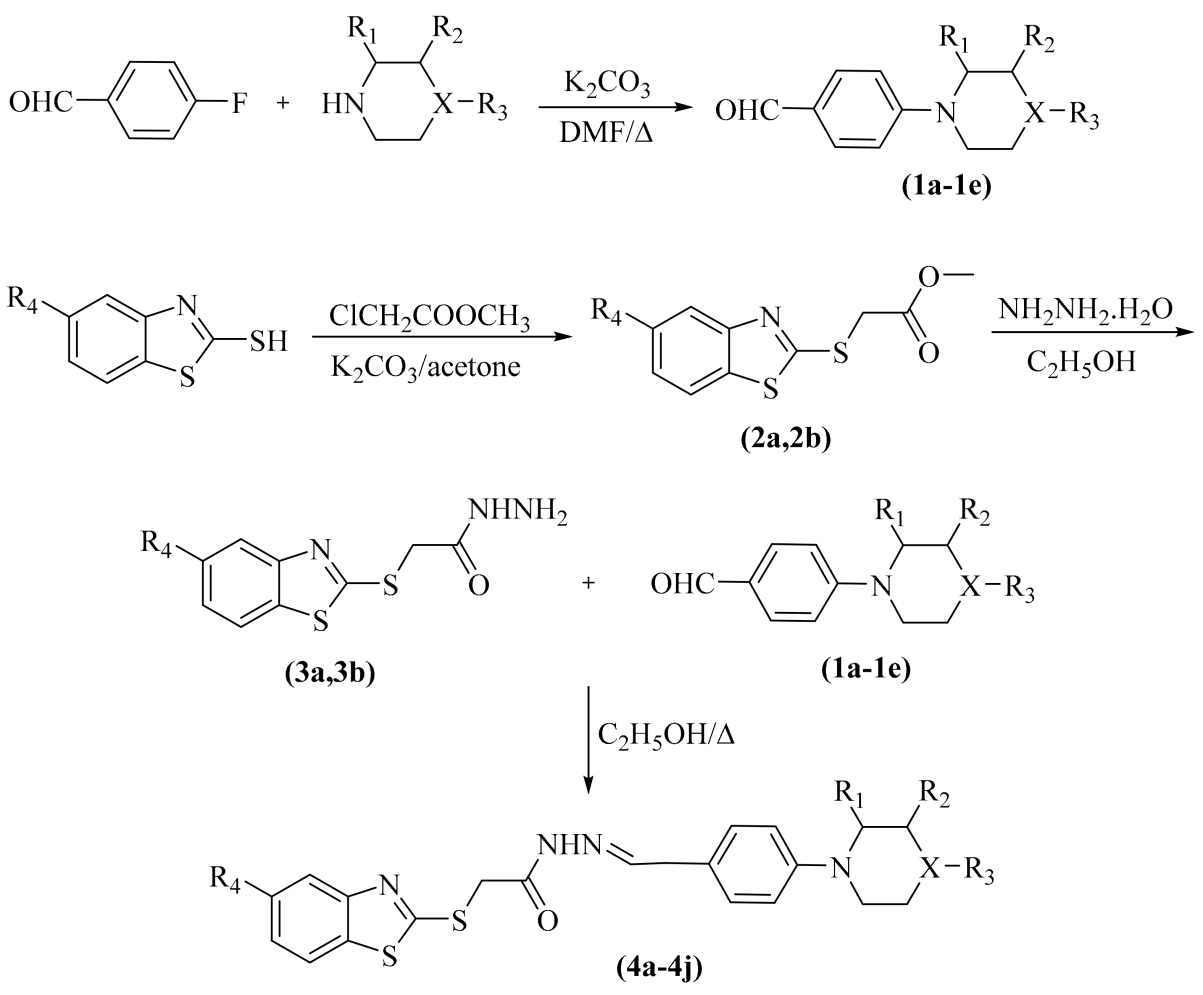
Synthesis way of the target compounds (**4a**–**4j**).

The compounds **4a**–**4j** were synthesized as summarized in Scheme 1. Initially, 4-substituted benzaldehydes (**1a**–**1e**) were synthesized under reflux by reaction of 4-fluorobenzaldehyde and appropriate secondary amine as reported previously [26]. Secondly, 5-substitutedbenzothiazole and ethyl chloroacetate were refluxed in acetone to obtain ethyl 2-(5-substitutedbenzothiazol-2-yl-thio)acetates (**2a**,**2b**), which reacted with excess of hydrazine hydrate to afford 2-((5-substitutedbenzothiazol-2-yl)thio)acetohydrazides (**3a,3b**) as described previously [27]. Finally, compounds **3a** and **3b** were reacted with appropriate 4-seconadaryaminesubstitutedbenzaldehydes (**1a**–**1e**) to gain the target compounds **4a**–**4j**.

Structure explanations of the final compounds were performed with IR, ^1^H-NMR, ^13^C-NMR, and HRMS spectroscopic methods (See Supplementary Materials). In the IR spectra of the compounds, stretching absorptions at 3165–3277 cm^−1^ proved N-H bond of the hydrazide groups. Signals belonging to carbonyl (C=O) function appeared at 1653–1664 cm^−1^. The stretching absorption at about 1193–1233 cm^−1^ were registered for C-N single bonds. The out of plane bending belonging to 1,4-disubstituted benzene was determined at 794–833 cm^−1^. In the ^1^H-NMR spectra, aliphatic protons belonging to piperidine and piperazine rings were recorded between 1.01 ppm and 4.20 ppm. Methyl groups on the piperidine ring gave doublet peaks between 0.89 ppm and 1.00 ppm. Methoxy substituents on benzothiazole and phenyl rings were observed as singlet peaks at 3.81 ppm and 3.69 ppm, respectively. Protons of methylene bridge were recorded as a singlet between 4.62 ppm and 4.66 ppm. Aromatic protons gave peaks between 6.84 ppm and 8.06 ppm. Peak splitting pattern of benzothiazole rings were recorded as two doublets with different coupling constant and one doublet of doublet, while phenyl rings gave two doublets with the same coupling constant. Protons on the azomethine moiety were observed as a singlet peak between 7.90 ppm and 7.95 ppm. N-H protons on the hydrazide moiety were recorded over 11.48 ppm. In ^13^C-NMR, aliphatic carbons were recorded between 13.37 ppm and 55.99 ppm. All aromatic carbons gave peaks from 105.03 to 159.17 ppm. C2 of benzothiazole was recorded around 162 ppm and carbonyl group gave peak over 168 ppm. In the HRMS spectra, all masses were matched well with the expected M + H values.

### 2.2. Biological Evaluation

#### 2.2.1. Cytotoxicity Test

MTT assay, depending on the capability of metabolically active cells that change the pale yellow MTT colour to a spectrophotometrically measured blue formazan salt, is one of the most favoured cytotoxicity tests [28]. We assessed the antitumor potential of new benzothiazole-acylhydrazones on A549, C6, MCF-7, and HT-29 cell lines with various concentrations (1 mM, 0.316 mM, 0.1 mM, 0.0316 mM, 0.01 mM, 0.00316 mM, 0.001 mM, 0.000316 mM). In addition, the cytotoxic activities of compounds **4a**–**4j** were evaluated against healthy NIH3T3 cells, owing to show the selectivity towards carcinogenic cells. The IC_50_ values of the compounds against cell lines are represented in Table 1.

Compound **4e** was found to have the highest cytotoxic activity against A549 cell lines. Moreover, the IC_50_ value of (0.03 mM) this compound against A549 cells was twofold lower than that of cisplatin (0.06 mM). However, the other compounds in the series could not display a significant cytotoxic activity against A549 cell lines.

**Figure 1:**
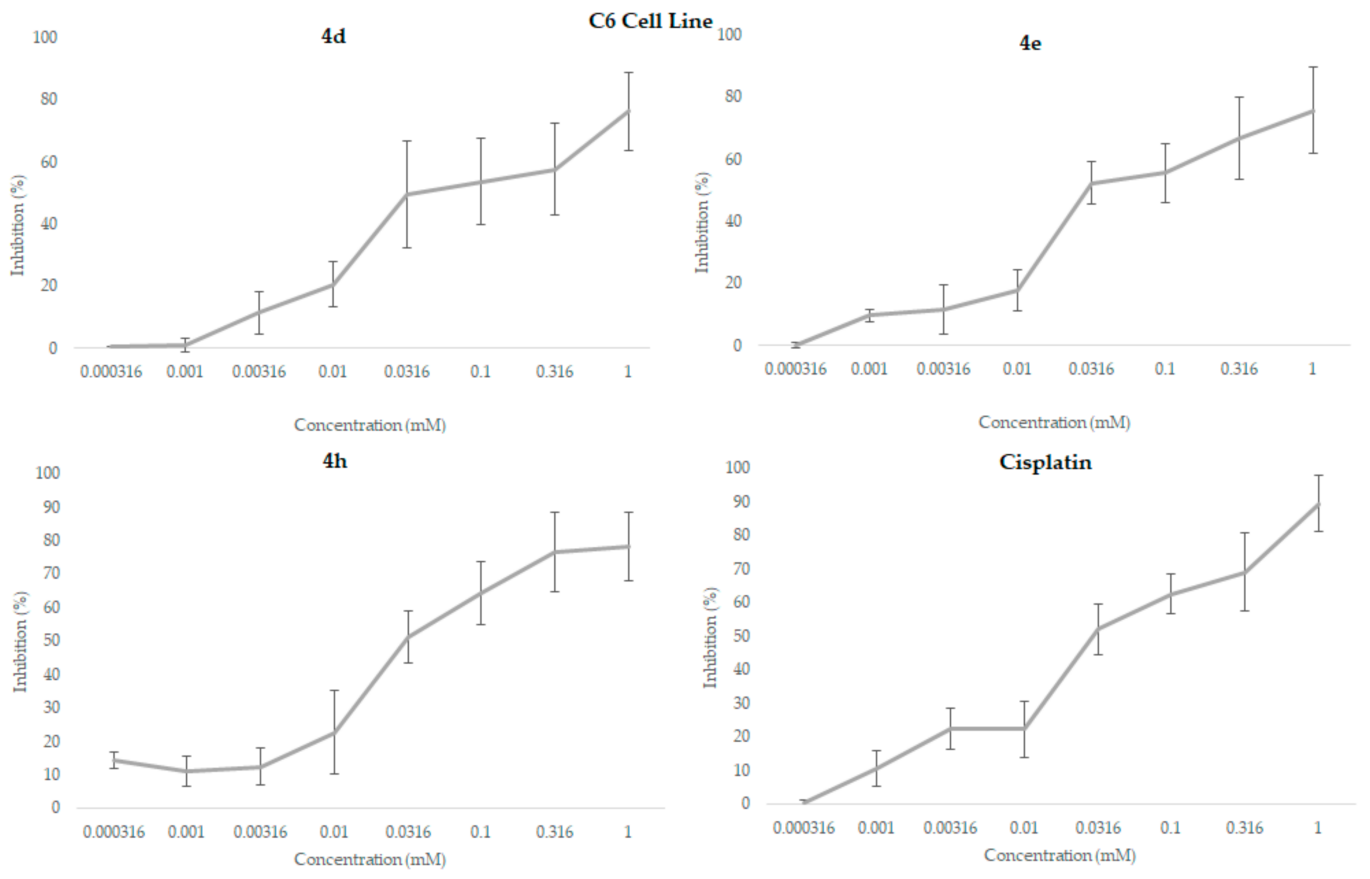
Inhibition values (%) of the compounds **4d**, **4e**, and **4h** and cisplatin for each concentration against C6 cell line following a 24 h incubation period in MTT assay. All data were expressed as mean ± standard deviation.

Compounds **4d**, **4e**, **4h** showed remarkable cytotoxicity against C6 cell lines. These compounds indicated equal IC_50_ value (0.03 mM) to cisplatin against C6 cell lines (Figure 1).

Compounds **4c** and **4d** were the most active compounds against both MCF-7 and HT-29 cell lines. However, these compounds indicated lower cytotoxic activity than the reference agent cisplatin.

In general, it has been determined that 5-chloro substitution of BT enhances the cytotoxic activity against carcinogenic cell lines. In addition, comparing 2, 3, or 4-methly substitution of piperidine indicated that three or four substituents makes more contribution to the cytotoxic activity against all carcinogenic cell lines.

One of the important criteria as to being anticancer agent candidates is to display minimum or no side-effects on healthy cells. Hence, cytotoxicity of synthesized compounds **4a**–**4j** against NIH3T3 cell line was determined. As seen in Table 1, compound **4e** showed higher cytotoxicity against NIH3T3 cell lines when compared to carcinogenic cell lines. On the other hand, IC_50_ values of compound **4h** against C6 cells were higher than its IC_50_ values against NIH3T3 cell lines. Moreover, compound **4d** indicated lower cytotoxicity against NIH3T3 cells when compared with both carcinogenic cell lines. This finding improved the importance of compound **4d** as a potential anticancer agent.

Comparing A549, C6, MCF-7, and HT-29 cell lines, the C6 cell line was found to be more susceptible to compounds. Therefore, the C6 cell line was used in further tests including DNA synthesis inhibition and flow cytometric analysis assays.

#### 2.2.2. DNA Synthesis Inhibition Assay

**Figure 2:**
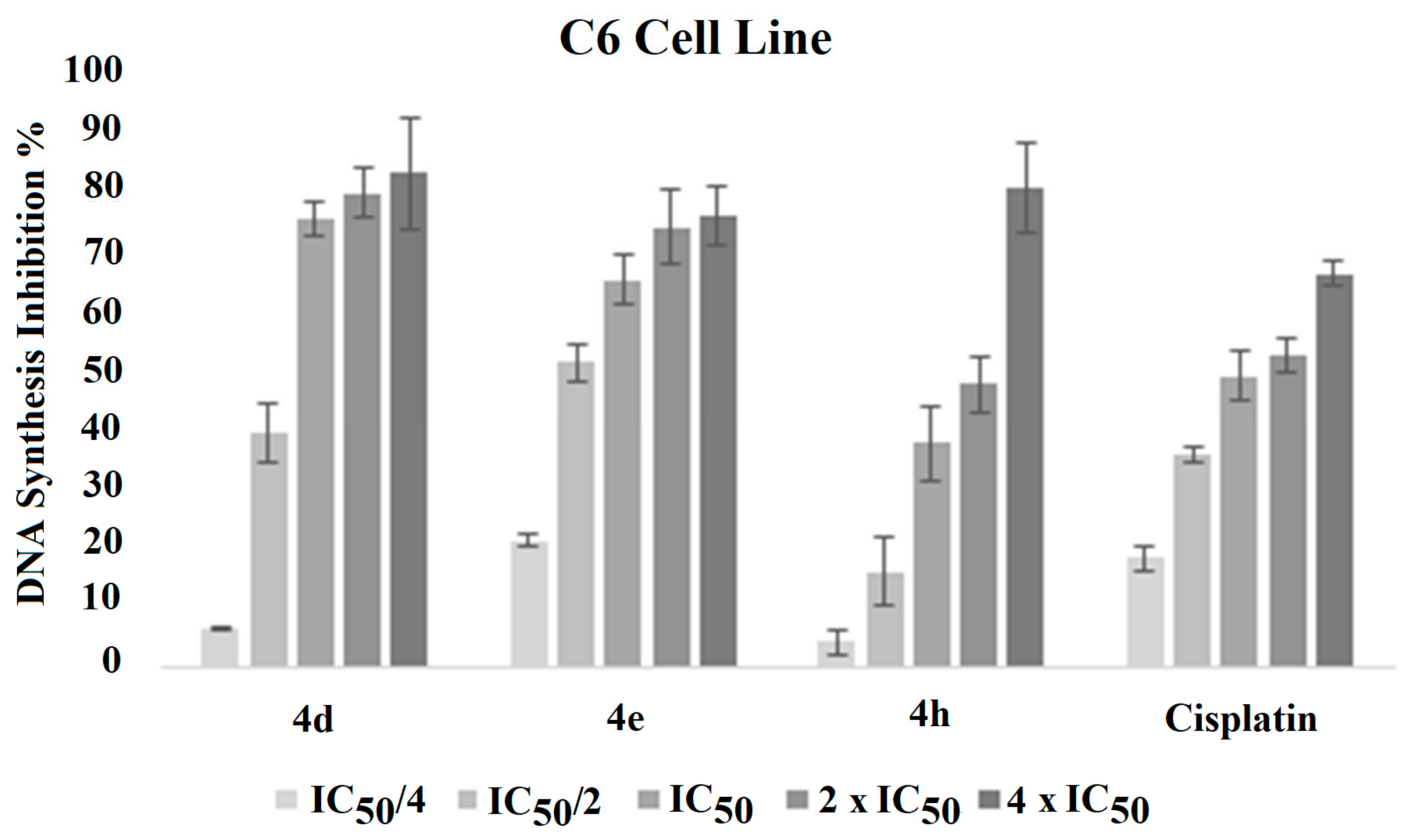
DNA synthesis inhibitory activity % of the compounds **4d**, **4e**, and **4h** and cisplatin against C6 cell line for 24 h incubation period.

This immunostaining assay is related to detecting the attendance of BrdU into nuclear DNA instead of thymidine during S-phase of the cell cycle using specific anti-BrdU antibodies [29,30]. Thus, this study was performed to evaluate inhibitory effects of compounds **4d**, **4e**, and **4h** on proliferation of C6 cell lines. Test compounds and reference agent cisplatin were used at the concentrations of IC_50_/4, IC_50_/2, IC_50_, 2 × IC_50_, and 4 × IC_50_. Figure 2 represents the DNA synthesis inhibitory effect of compounds **4d**, **4e**, and **4h** on C6 cells.

Tested compounds exhibited concentration-dependent inhibitory activity on DNA synthesis of C6 cell lines. When compared the DNA synthesis inhibitory activity of compounds, it is clear that **4d** has higher inhibition potency than cisplatin at all concentrations. Compound **4e** also displayed a higher antiproliferative effect than cisplatin at the concentrations of IC_50_/2, IC_50_, 2 × IC_50_, and 4 × IC_50_. However, compound **4h** could not display the same ability as compounds **4d** and **4e** to inhibit DNA synthesis. According to these results, it can be suggested that compound **4d** possesses a higher antiproliferative activity against C6 cell lines than cisplatin.

#### 2.2.3. Flow Cytometric Analysis

In the therapy of cancer, apoptosis, or programmed cell death, is one of the distinguished mechanisms of cytotoxic agents. It is an important approach to express the cell death pathway and most of the anticancer drugs induce apoptosis in tumor cells. Annexin V-FITC is a reagent that can detect and measure quantitate of apoptotic cells by flow cytometry. Staining cells with PI and Annexin V-FITC show the differences between live, apoptotic, dead, and late apoptotic or necrotic cells [31]. Thus, this method provides evidence on the pathway of cell death.

Flow cytometry studies were performed on C6 cells for compounds **4d**, **4e**, and **4h** and cisplatin with the aim of determining the stimulated cellular death pathway. Selected compounds and cisplatin were used at the concentrations of IC_50_/2, IC_50_, and 2 × IC_50_. The flow cytometric analysis results of compounds **4d**, **4e**, and **4h** and cisplatin for C6 cell line are represented in Table 2.

**Figure 3:**
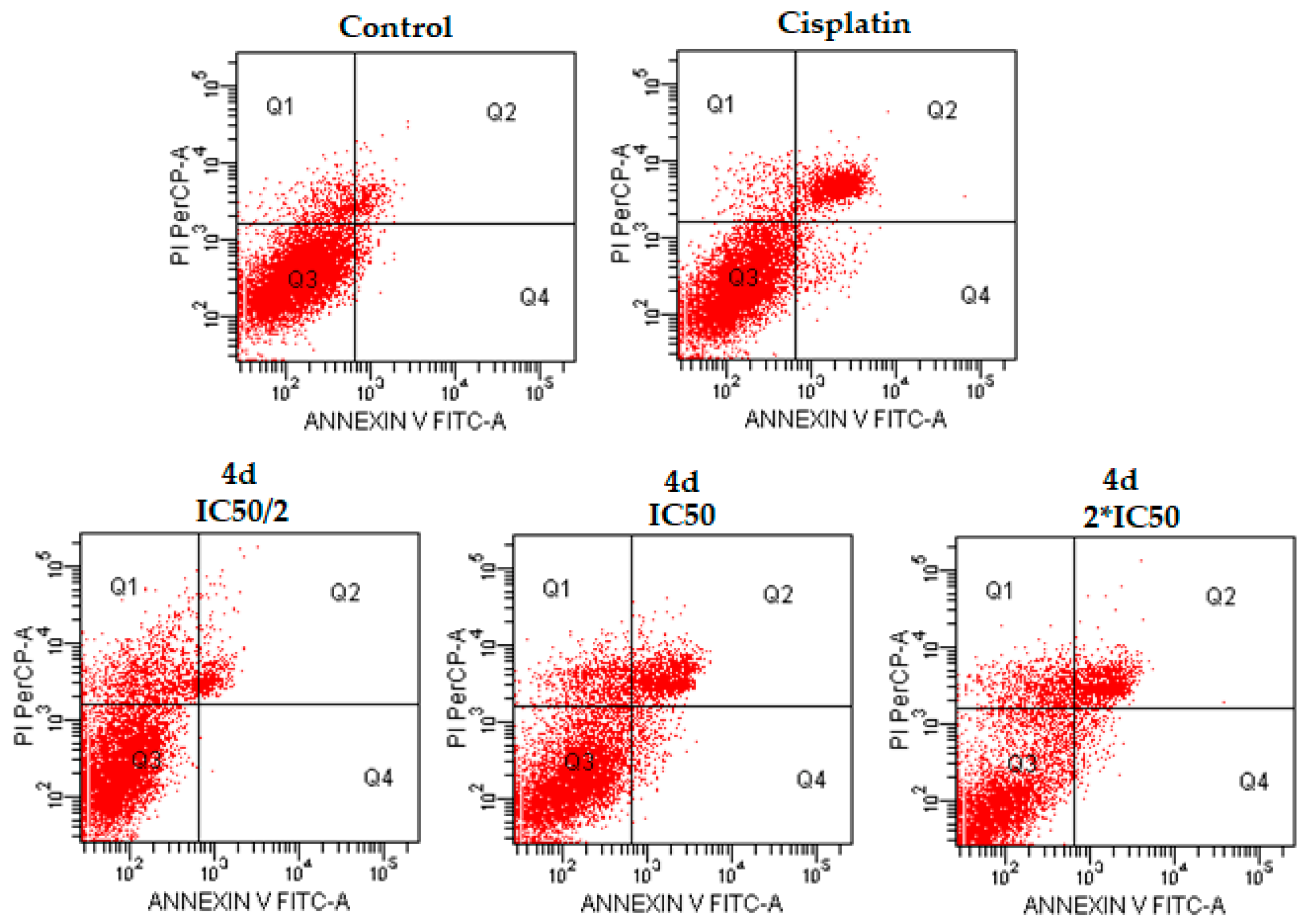
The flow cytometric analysis diagram of compound **4d** and cisplatin for C6 cell line. Annexin V-PI analysis in C6 cell line, following 24 h incubation period of compounds **4d** at IC_50_/2, IC_50_, 2 × IC_50_ concentrations, which are 0.015, 0.03 and 0.06 mM, respectively, following 24 h incubation period. FITC Annexin V method in C6 cell line, following a 24 h incubation period of cisplatin at IC_50_ (0.03 mM). A diagram can be divided into four regions that are defined as follows: the percentage of necrotic cells (PI/FITC. +/−; Q1); the percentage of late apoptotic cells (Q2; PI/FITC +/+); the percentage of viable cells (Q3; PI/FITC −/−) and the percentage of early apoptotic cells (Q4; PI/FITC −/+). Percentages of Q1, Q2, Q3, and Q4 of compound **4d** and cisplatin were presented in Table 2.

**Figure 4:**
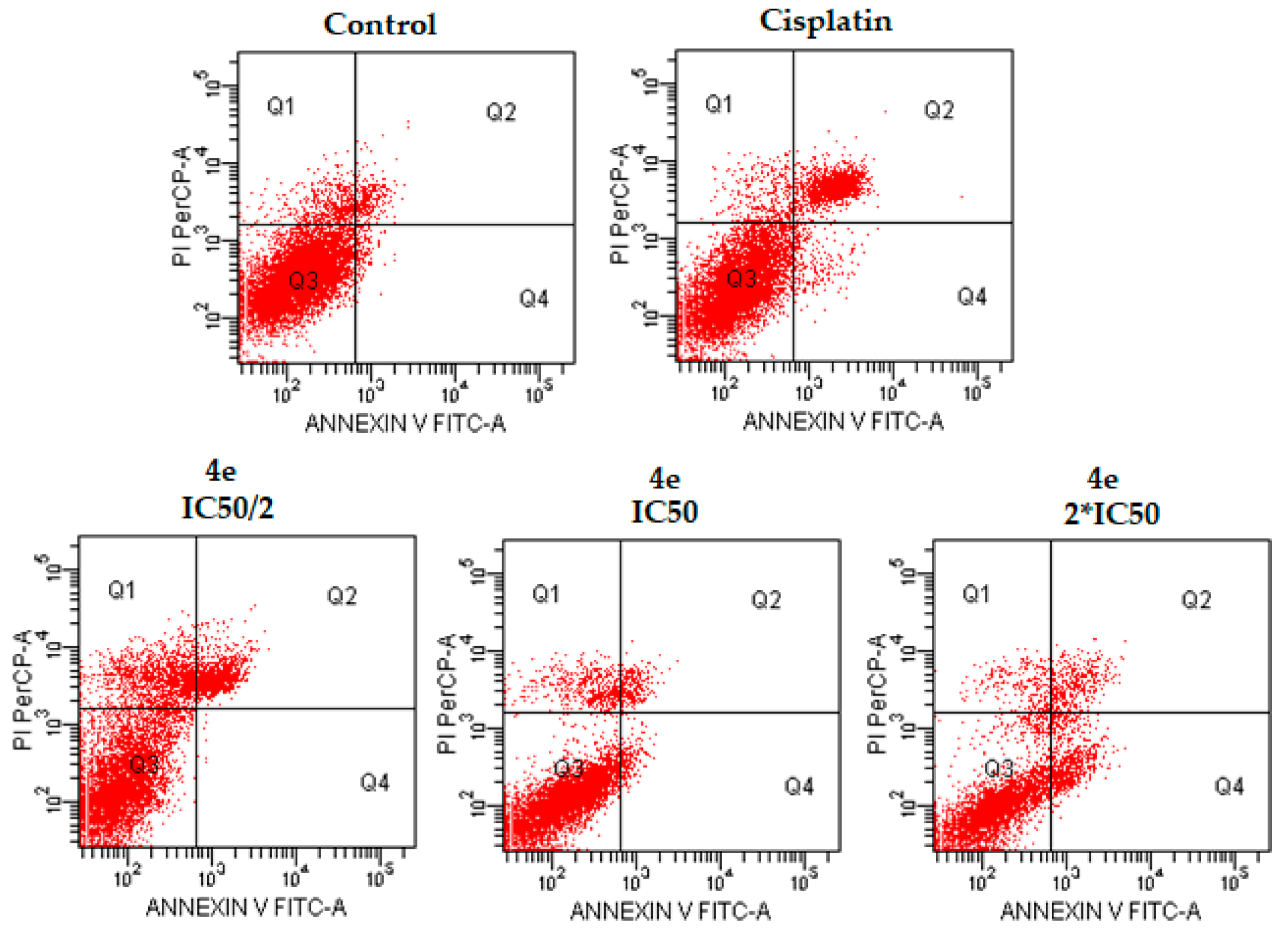
The flow cytometric analysis diagram of compound **4e** and cisplatin for C6 cell line. Annexin V-PI analysis in C6 cell line, following a 24 h incubation period of compounds **4e** at IC_50_/2, IC_50_, 2 × IC_50_ concentrations, which are 0.015, 0.03 and 0.06 mM, respectively, following a 24 h incubation period. An FITC Annexin V method in C6 cell line, following a 24 h incubation period of cisplatin at IC_50_ (0.03 mM). A diagram can be divided into four regions that are defined as follows: the percentage of necrotic cells (PI/FITC. +/−; Q1); the percentage of late apoptotic cells (Q2; PI/FITC +/+); the percentage of viable cells (Q3; PI/FITC −/−) and the percentage of early apoptotic cells (Q4; PI/FITC −/+). Percentages of Q1, Q2, Q3, and Q4 of compound **4e** and cisplatin were presented in Table 2.

**Figure 5:**
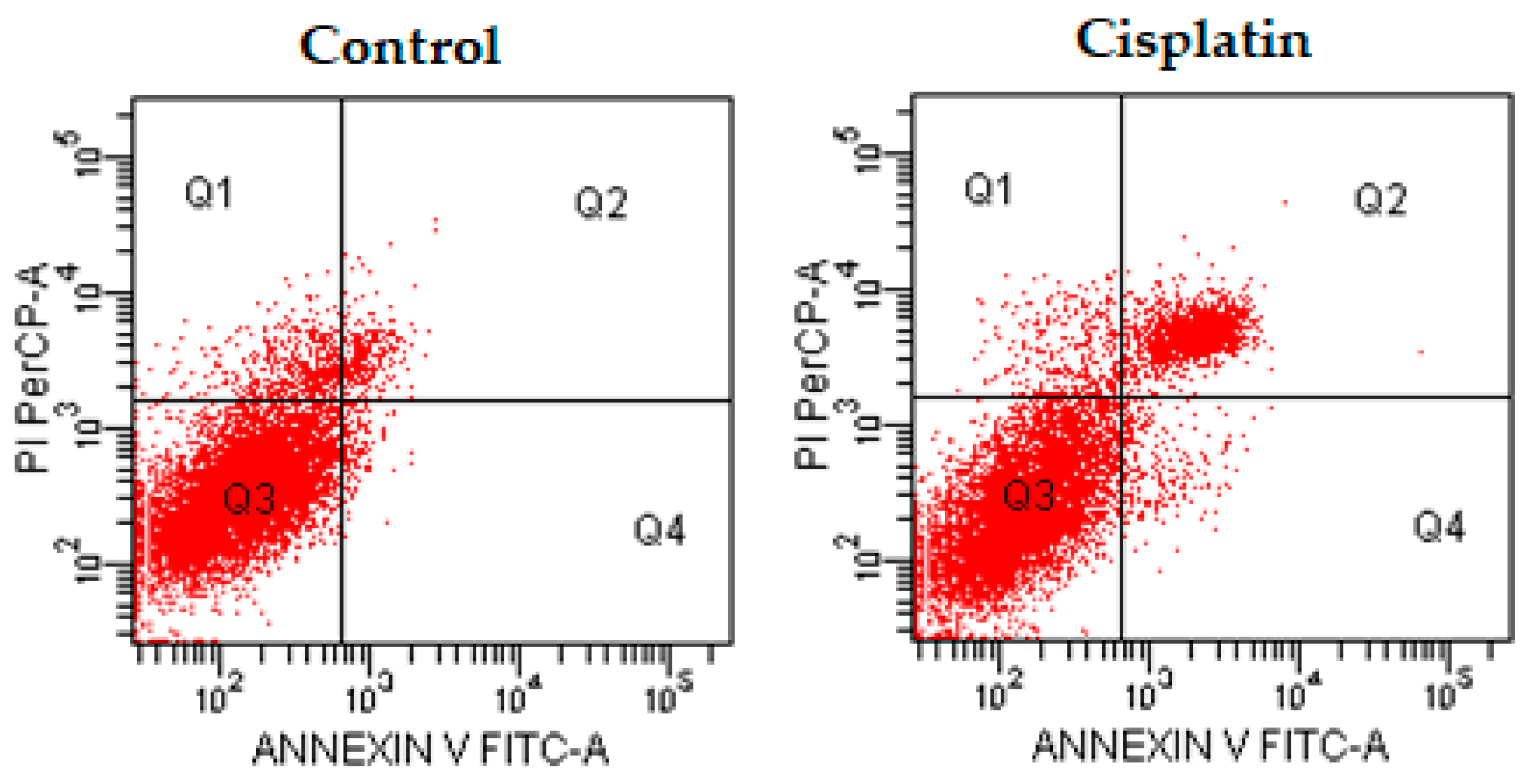

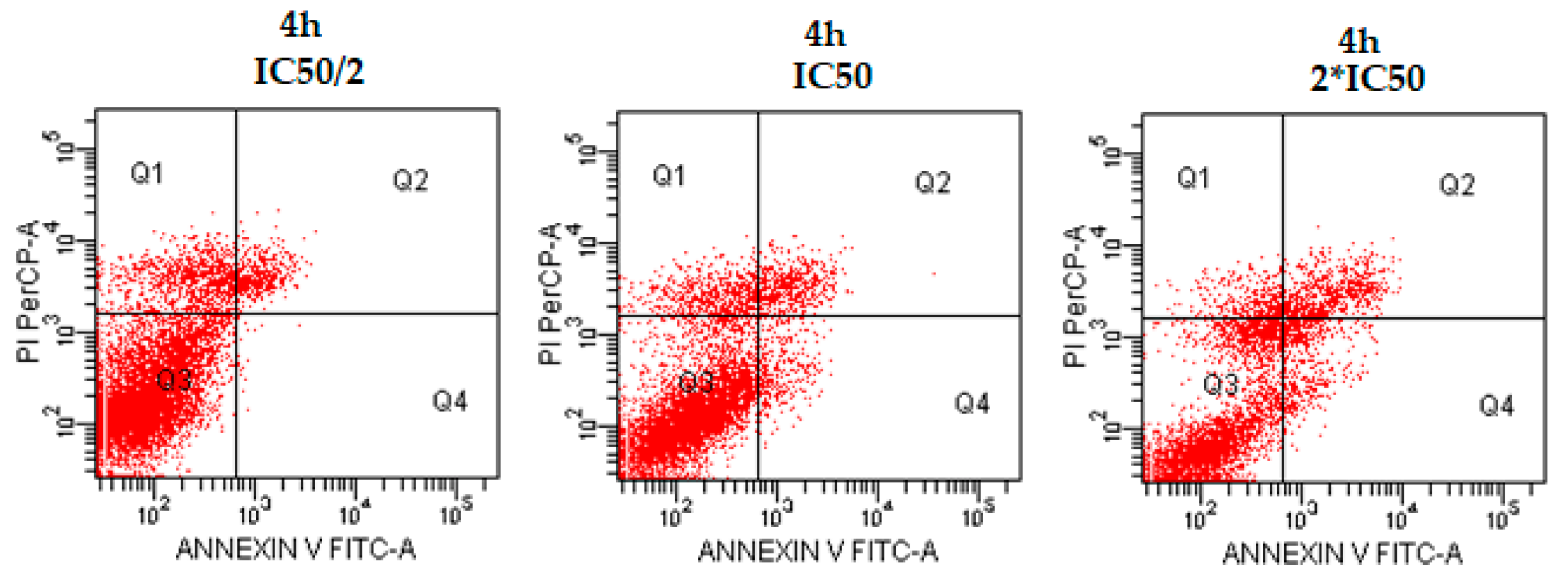
The flow cytometric analysis diagram of compound **4h** and cisplatin for C6 cell line. Annexin V-PI analysis in C6 cell line, following 24 h incubation period of compounds **4h** at IC_50_/2, IC_50_, 2 × IC_50_ concentrations, which are 0.015, 0.03 and 0.06 mM, respectively, following a 24 h incubation period. FITC Annexin V method in C6 cell line, following 24 h incubation period of cisplatin at IC_50_ (0.03 mM). Diagram can be divided into four regions that are defined as follows: the percentage of necrotic cells (PI/FITC. +/−; Q1); the percentage of late apoptotic cells (Q2; PI/FITC +/+); the percentage of viable cells (Q3; PI/FITC −/−) and the percentage of early apoptotic cells (Q4; PI/FITC −/+). Percentages of Q1, Q2, Q3, and Q4 of compound **4h** and cisplatin were presented in Table 2.

Additionally, flow cytometric analysis diagrams are presented in Figure 3, Figure 4 and Figure 5. As seen in the figures, compounds **4d**, **4e**, and **4h** exhibited comparable population of apoptotic cells to cisplatin. The percentages of apoptotic cells were determined as 5.5, 19.5, and 12.0% for compounds **4d**, **4e**, and **4h**, respectively, at IC_50_ concentrations, whereas the percentage of apoptotic cells was determined as 20.9% for cisplatin at IC_50_ concentration. Compound **4e** showed the most apoptotic activity (19.5%) when compared with other compounds at IC_50_ concentrations (Table 2). While compound **4d** induced necrotic cell death in IC_50_/2 and IC_50_ concentrations, especially IC_50_/2 concentrations, compounds **4e** and **4h** induced necrotic cell death at IC_50_/2 concentration. At this point, we could emphasise that compound **4d**, **4e** and **4h** induced apoptotic cell death, dose-dependently.

## 3. Materials and Methods

### 3.1. Chemistry

All chemicals in this study were obtained either from Sigma-Aldrich (Sigma-Aldrich Corp., St. Louis, MO, USA) or Merck (Merck KGaA, Darmstadt, Germany), and used without further chemical purifications. Melting points of the compounds were measured by using an automatic melting point determination instrument (MP90, Mettler-Toledo, Columbus, OH, USA) and were presented as uncorrected. ^1^H- and ^13^C-NMR spectra were recorded in DMSO-*d*_6_ by a Bruker digital FT-NMR spectrometer (Bruker Bioscience, Billerica, MA, USA) at 300 MHz and 75 MHz, respectively. The IR spectra of the compounds were recorded using an IRAffinity-1S Fourier transform IR (FTIR) spectrometer (Shimadzu, Tokyo, Japan). HRMS studies were performed on an LCMS-IT-TOF system (Shimadzu, Tokyo, Japan). Chemical purities of the compounds were checked by classical TLC applications performed on silica gel 60 F_254_ (Merck KGaA, Darmstadt, Germany).

#### 3.1.1. General Synthesis Procedure for 4-Substitutebenzaldehyde Derivatives (**1a**–**1e**)

Appropriate secondary amine (0.007 mol), 4-fluorobenzaldehyde (0.007 mol, 0.75 mL) and K_2_CO_3_ (0.007 mol, 0.96 g) were refluxed in DMF (10 mL) for 10 h. The mixture was cooled with ice-water (50 mL), a precipitated product was filtered, washed with deionised water, dried, and recrystallized from EtOH to give compounds **1a**–**1e** in the yields range of 63 to 72%.

#### 3.1.2. General Procedure for Ethyl 2-(5-Substitutedbenzothiazol-2-yl-thio)acetate (**2a**–**2b**)

Corresponding 5-substitutedbenzothiazol-2-thiol (0.015 mol), ethyl 2-chloroacetate, (0.015 mol, 1.59 g), and K_2_CO_3_ (0.015 mol, 2.07 g) were refluxed in acetone for 2h. The solvent was removed under reduced pressure and the residue was recrystallized from EtOH to give compounds **2a** and **2b** with yields of 78% and 81%, respectively.

#### 3.1.3. General Procedure for 2-((5-Substitutedbenzothiazol-2-yl)thio)acetohydrazide (**3a**–**3b**)

Hydrazine hydrate (5 mL) was added into EtOHic solution (20 mL) of 5-substituted ethyl 2-((benzothiazol-2-yl)thio)acetate (**2a**–**2b**) (0.012 mol) in portions with constant stirring. Then, the mixture was stirred for 3 h. The precipitated product was filtered, dried and recrystallized from EtOH to give compounds **3a** and **3b** with yields of 84% and 82%, respectively.

#### 3.1.4. General Synthesis Procedure of Compounds (**4a**–**4j**)

Appropriate 2-((5-substitutedbenzothiazol-2-yl)thio) acetohydrazide (**3a**–**3b**) (0.002 mol), 4-substitutedbenzaldehyde (0.002 mol) and glacial acetic acid (0.1 mL) were refluxed in EtOH for 2 h. The mixture was cooled down in an ice-bath, precipitated product was filtered, dried and recrystallized from EtOH.

*2-((5-Chlorobenzothiazol-2-yl)thio)-N*’*-(4-(piperidin-1-yl)benzylidene)acetohydrazide* (**4a**). Yield: 83%, M.P. = 178–179 °C, FTIR (ATR, cm^−1^): 3277 (N-H), 1654 (C=O), 1232 (C-N), 1022, 813, 794. ^1^H-NMR (300 MHz, DMSO-*d*_6_): δ = 1.57 (6H, br.s, piperidine), 3.25 (4H, br.s, piperidine), 4.66 (2H, s, -CH_2_-), 6.91 (2H, d, *J* = 8.86 Hz, Ar-H), 7.42 (1H, dd, *J*_1_ = 2.10 Hz, *J*_2_ = 8.55 Hz, BT-H), 7.49 (2H, d, *J* = 8.86 Hz, Ar-H), 7.91 (1H, s, -CH=N-), 7.93 (1H, d, *J* = 2.10 Hz, BT-H), 8.05 (1H, d, *J* = 8.55 Hz, BT-H), 11.52 (1H, s, -NH). ^13^C-NMR (75 MHz, DMSO-*d*_6_): δ = 24.4, 25.4, 35.8, 48.9, 115.0, 121.1, 123.7, 124.9, 128.6, 131.7, 134.0, 144.9, 148.2, 152.8, 153.9, 162.7, 168.1. HRMS (*m*/*z*): [M + H]^+^ calcd for C_21_H_21_N_4_OS_2_Cl: 445.0918; found: 445.0905.

*2-((5-Chlorobenzothiazol-2-yl)thio)-N*’*-(4-(2-methylpiperidin-1-yl)benzylidene)acetohydrazide* (**4b**). Yield: 79%, M.P. = 138–139 °C, FTIR (ATR, cm^−1^): 3255 (N-H), 1654 (C=O), 1193 (C-N), 1068, 813, 794. ^1^H-NMR (300 MHz, DMSO-*d*_6_): δ = 1.00 (3H, d, *J* = 6.63 Hz, -CH_3_), 1.45–1.56 (4H, m, piperidine), 1.70–1.74 (2H, m, piperidine), 2.83–2.91 (1H, m, piperidine), 3.42–3.49 (1H, m, piperidine), 4.19–4.20 (1H, m, piperidine), 4.66 (2H, s, -CH_2_-), 6.87 (2H, d, *J* = 8.80 Hz, Ar-H), 7.42 (1H, dd, *J*_1_ = 2.10 Hz, *J*_2_ = 8.55 Hz, BT-H), 7.47 (2H, d, *J* = 8.80 Hz, Ar-H), 7.91 (1H, s, -CH=N-), 7.93 (1H, d, *J* = 2.10 Hz, BT-H), 8.05 (1H, d, *J* = 8.55 Hz, BT-H), 11.49 (1H, s, -NH). ^13^C-NMR (75 MHz, DMSO-*d*_6_): δ = 13.4, 18.7, 25.8, 30.8, 35.8, 41.8, 49.2, 114.9, 121.1, 123.7, 124.9, 128.7, 131.7, 134.0, 144.9, 148.3, 152.2, 153.9, 162.7, 168.1. HRMS (*m*/*z*): [M + H]^+^ calcd for C_22_H_23_N_4_OS_2_Cl: 459.1075; found: 459.1067.

*2-((5-Chlorobenzothiazol-2-yl)thio)-N*’*-(4-(3-methylpiperidin-1-yl)benzylidene)acetohydrazide* (**4c**). Yield: 81%, M.P. = 149–150 °C, FTIR (ATR, cm^−1^): 3240 (N-H), 1654 (C=O), 1230 (C-N), 1022, 792. ^1^H-NMR (300 MHz, DMSO-*d*_6_): δ = 0.91 (3H, d, *J* = 6.51 Hz, -CH_3_), 1.01–1.13 (1H, m, piperidine), 1.45–1.77 (4H, m, piperidine), 2.36–2.43 (1H, m, piperidine), 2.65–2.74 (1H, m, piperidine), 3.68–3.75 (2H, m, piperidine), 4.66 (2H, s, -CH_2_-), 6.90 (2H, d, *J* = 8.88 Hz, Ar-H), 7.41 (1H, dd, *J*_1_ = 2.10 Hz, *J*_2_ = 8.55 Hz, BT-H), 7.47 (2H, d, *J* = 8.88 Hz, Ar-H), 7.90 (1H, s, -CH=N-), 7.93 (1H, d, *J* = 2.10 Hz, BT-H), 8.05 (1H, d, *J* = 8.55 Hz, BT-H), 11.51 (1H, s, -NH). ^13^C-NMR (75 MHz, DMSO-*d*_6_): δ = 19.7, 24.8, 30.5, 32.9, 35.8, 48.3, 55.7, 114.9, 121.1, 123.7, 124.9, 128.7, 131.7, 134.0, 144.9, 148.2, 152.6, 153.9, 162.7, 168.1. HRMS (*m*/*z*): [M + H]^+^ calcd for C_22_H_23_N_4_OS_2_Cl: 459.1075; found: 459.1063.

*2-((5-Chlorobenzothiazol-2-yl)thio)-N*’*-(4-(4-methylpiperidin-1-yl)benzylidene)acetohydrazide* (**4d**). Yield: 85%, M.P. = 153–154 °C, FTIR (ATR, cm^−1^): 3255 (N-H), 1660 (C=O), 1226 (C-N), 1022, 817, 792. ^1^H-NMR (300 MHz, DMSO-*d*_6_): δ = 0.90 (3H, d, *J* = 6.45 Hz, -CH_3_), 1.09–1.22 (2H, m, piperidine), 1.48–1.55 (1H, m, piperidine), 1.63–1.67 (2H, m, piperidine), 2.68–2.75 (2H, m, piperidine), 3.74–3.79 (2H, m, piperidine), 4.66 (2H, s, -CH_2_-), 6.89 (2H, d, *J* = 8.90 Hz, Ar-H), 7.40 (1H, dd, *J*_1_ = 2.05 Hz, *J*_2_ = 8.55 Hz, BT-H), 7.48 (2H, d, *J* = 8.90 Hz, Ar-H), 7.89 (1H, d, *J* = 2.05 Hz, BT-H), 7.91 (1H, s, -CH=N-), 8.03 (1H, d, *J* = 8.55 Hz, BT-H), 11.53 (1H, s, -NH). ^13^C-NMR (75 MHz, DMSO-*d*_6_): δ = 22.2, 30.7, 33.7, 35.8, 48.2, 115.0, 121.1, 123.7, 124.9, 128.6, 131.7, 134.0, 144.9, 148.2, 152.6, 153.9, 162.7, 168.1. HRMS (*m*/*z*): [M + H]^+^ calcd for C_22_H_23_N_4_OS_2_Cl: 459.1075; found: 459.1062.

*2-((5-Chlorobenzothiazol-2-yl)thio)-N*’*-(4-(4-(4-methoxyphenyl)piperazin-1-yl)benzylidene)acetohydrazide* (**4e**). Yield: 78%, M.P. = 211–212 °C, FTIR (ATR, cm^−1^): 3201 (N-H), 1653 (C=O), 1226 (C-N), 1031, 825, 721. ^1^H-NMR (300 MHz, DMSO-*d*_6_): δ = 3.14 (4H, br.s, piperazine), 3.38 (4H, br.s, piperazine), 3.69 (3H, s, -OCH_3_), 4.68 (2H, s, -CH_2_-), 6.85 (2H, d, *J* = 9.09 Hz, Ar-H), 6.96 (2H, d, *J* = 9.09 Hz, Ar-H), 7.04 (2H, d, *J* = 8.76 Hz, Ar-H), 7.42 (1H, dd, *J*_1_ = 2.10 Hz, *J*_2_ = 8.55 Hz, BT-H), 7.55 (2H, d, *J* = 8.76 Hz, Ar-H), 7.94 (1H, d, *J* = 2.10 Hz, BT-H), 7.95 (1H, s, -CH=N-), 8.05 (1H, d, *J* = 8.55 Hz, BT-H), 11.57 (1H, s, -NH). ^13^C-NMR (75 MHz, DMSO-*d*_6_): δ = 35.8, 47.9, 50.1, 55.6, 114.8, 115.2, 118.2, 121.1, 123.7, 124.9, 128.6, 128.9, 131.7, 134.0, 144.8, 145.7, 148.1, 152.4, 153.7, 162.8, 168.2. HRMS (*m*/*z*): [M + H]^+^ calcd for C_27_H_26_N_5_O_2_S_2_Cl: 552.1289; found: 552.1293.

*2-((5-Methoxybenzothiazol-2-yl)thio)-N*’*-(4-(piperidin-1-yl)benzylidene)acetohydrazide* (**4f**). Yield: 82%, M.P. = 138–139 °C, FTIR (ATR, cm^−1^): 3207 (N-H), 1664 (C=O), 1230 (C-N), 1031, 810, 721. ^1^H-NMR (300 MHz, DMSO-*d*_6_): δ = 1.56 (6H, br.s., piperidine), 3.23 (4H, br.s., piperidine), 3.81 (3H, s, -OCH_3_), 4.63 (2H, s, -CH_2_-), 6.90 (2H, d, *J* = 8.95 Hz, Ar-H), 6.99 (1H, dd, *J*_1_ = 2.50 Hz, *J*_2_ = 8.80 Hz), 7.41 (1H, d, *J* = 2.50 Hz, BT-H), 7.48 (2H, d, *J* = 8.95 Hz, Ar-H), 7.87 (1H, d, *J* = 8.80 Hz, BT-H), 7.93 (1H, s, -CH=N-), 11.51 (1H, s, -NH). ^13^C-NMR (75 MHz, DMSO-*d*_6_): δ = 24.4, 25.4, 35.6, 48.9, 55.9, 105.0, 114.2, 115.1, 122.5, 123.5, 128.6, 144.8, 148.1, 152.8, 154.4, 159.2, 162.9, 168.2. HRMS (*m*/*z*): [M + H]^+^ calcd for C_22_H_24_N_4_O_2_S_2_: 441.1413; found: 441.1408.

*2-((5-Methoxybenzothiazol-2-yl)thio)-N*’*-(4-(2-methylpiperidin-1-yl)benzylidene)acetohydrazide* (**4g**). Yield: 79%, M.P. = 138–139 °C, FTIR (ATR, cm^−1^): 3267 (N-H), 1664 (C=O), 1230 (C-N), 1030, 824, 794. ^1^H-NMR (300 MHz, DMSO-*d*_6_): δ = 1.02 (3H, d, *J* = 6.60 Hz, -CH_3_), 1.47–1.58 (4H, m, piperidine), 1.73–1.78 (2H, m, piperidine), 2.83–2.91 (1H, m, piperidine), 3.42–3.49 (1H, m, piperidine), 3.81 (3H, s, -OCH_3_), 4.19–4.20 (1H, m, piperidine), 4.66 (2H, s, -CH_2_-), 6.87 (2H, d, *J* = 8.80 Hz, Ar-H), 6.99 (1H, dd, *J*_1_ = 2.50 Hz, *J*_2_ = 8.80 Hz), 7.41 (1H, d, *J* = 2.50 Hz, BT-H), 7.48 (2H, d, *J* = 8.95 Hz, Ar-H), 7.90 (1H, d, *J* = 8.80 Hz, BT-H), 7.92 (1H, s, -CH=N-), 11.50 (1H, s, -NH). ^13^C-NMR (75 MHz, DMSO-*d*_6_): δ = 13.4, 18.7, 25.8, 30.8, 35.8, 41.8, 49.8, 55.9, 105.1, 114.8, 115.5, 122.6, 123.5, 128.7, 144.4, 148.1, 152.3, 154.8, 159.2, 162.7, 168.1. HRMS (*m*/*z*): [M + H]^+^ calcd for C_23_H_26_N_4_O_2_S_2_: 455.1570; found: 455.1564.

*2-((5-Methoxybenzo[d]thiazol-2-yl)thio)-N*’*-(4-(3-methylpiperidin-1-yl)benzylidene)acetohydrazide* (**4h**). Yield: 81%, M.P. = 147–148 °C, FTIR (ATR, cm^−1^): 3165 (N-H), 1660 (C=O), 1228 (C-N), 1029, 808, 783. ^1^H-NMR (300 MHz, DMSO-*d*_6_): δ = 0.89 (3H, d, *J* = 6.54 Hz, -CH_3_), 1.03–1.08 (1H, m, piperidine), 1.48–1.76 (4H, m, piperidine), 2.34–2.42 (1H, m, piperidine), 2.64–2.72 (1H, m, piperidine), 3.66–3.73 (2H, m, piperidine), 3.81 (3H, s, -OCH_3_), 4.63 (2H, s, -CH_2_-), 6.89 (2H, d, *J* = 8.77 Hz, Ar-H), 6.99 (1H, dd, *J*_1_ = 2.46 Hz, *J*_2_ = 8.80 Hz BT-H), 7.41 (1H, d, *J* = 2.46 Hz), 7.47 (2H, d, *J* = 8.77 Hz, Ar-H), 7.85 (1H, d, *J* = 8.80 Hz, BT-H), 7.93 (1H, s, -CH=N-), 11.52 (1H, s, -NH). ^13^C-NMR (75 MHz, DMSO-*d*_6_): δ = 19.6, 24.8, 30.5, 32.9, 35.6, 48.3, 55.7, 105.0, 114.2, 114.9, 122.5, 123.4, 128.6, 144.8, 148.1, 152.5, 154.4, 159.2, 162.9, 168.2. HRMS (*m*/*z*): [M + H]^+^ calcd for C_23_H_26_N_4_O_2_S_2_: 455.1570; found: 455.1569.

*2-((5-Methoxybenzo[d]thiazol-2-yl)thio)-N*’*-(4-(4-methylpiperidin-1-yl)benzylidene)acetohydrazide* (**4i**). Yield: 79%, M.P. = 149–150 °C, FTIR (ATR, cm^−1^): 3215 (N-H), 1664 (C=O), 1228 (C-N), 1031, 833, 804. ^1^H-NMR (300 MHz, DMSO-*d*_6_): δ = 0.92 (3H, d, *J* = 6.39 Hz, -CH_3_), 1.03–1.08 (1H, m, piperidine), 1.11–1.23 (2H, m, piperidine), 1.52–1.57 (1H, m, piperidine), 1.64–1.69 (2H, m, piperidine), 2.69–2.77 (2H, m, piperidine), 3.75 (1H, br.s., piperidine), 3.81 (3H, s, -OCH_3_), 4.62 (2H, s, -CH_2_-), 6.91 (2H, d, *J* = 8.70 Hz, Ar-H), 6.99 (1H, dd, *J*_1_ = 2.30 Hz, *J*_2_ = 8.80 Hz, BT-H), 7.40 (1H, d, *J* = 2.30 Hz), 7.48 (2H, d, *J* = 8.70 Hz, Ar-H), 7.85 (1H, d, *J* = 8.80 Hz, BT-H), 7.92 (1H, s, -CH=N-), 11.50 (1H, s, -NH). ^13^C-NMR (75 MHz, DMSO-*d*_6_): δ = 22.2, 30.7, 33.7, 35.6, 48.2, 55.9, 105.0, 114.2, 115.1, 122.5, 123.5, 128.6, 144.8, 148.1, 152.5, 154.4, 159.2, 162.9, 168.2. HRMS (*m*/*z*): [M + H]^+^ calcd for C_23_H_26_N_4_O_2_S_2_: 455.1570; found: 455.1566.

*2-((5-Methoxybenzo[d]thiazol-2-yl)thio)-N*’*-(4-(4-(4-methoxyphenyl)piperazin-1-yl)benzylidene)acetohydrazide* (**4j**). Yield: 80%, M.P. = 199–200 °C, FTIR (ATR, cm^−1^): 3226 (N-H), 1656 (C=O), 1224 (C-N), 1029, 812, 715. ^1^H-NMR (300 MHz, DMSO-*d*_6_): δ = 3.14 (4H, br.s., piperazine), 3.39 (4H, br.s., piperazine), 3.69 (3H, s, -OCH_3_), 3.81 (3H, s, -OCH_3_), 4.64 (2H, s, -CH_2_-), 6.84 (2H, d, *J* = 9.15 Hz, Ar-H), 6.96 (2H, d, *J* = 9.15 Hz), 6.97–7.05 (3H, m, Ar-H, BT-H), 7.41 (1H, d, *J* = 2.46 Hz, BT-H), 7.55 (2H, d, *J* = 8.85 Hz, Ar-H), 7.88 (1H, d, *J* = 8.79 Hz, BT-H), 7.95 (1H, s, -CH=N-), 11.54 (1H, s, -NH). ^13^C-NMR (75 MHz, DMSO-*d*_6_): δ = 35.6, 47.9, 50.1, 55.7, 55.9, 105.0, 114.2, 114.8, 115.2, 118.2, 122.5, 124.5, 128.6, 144.7, 145.7, 147.9, 152.4, 153.7, 154.4, 159.2, 162.9, 168.3. HRMS (*m*/*z*): [M + H]^+^ calcd for C_28_H_29_N_5_O_3_S_2_: 548.1785; found: 548.1783.

### 3.2. Biological Evaluation

#### 3.2.1. Cytotoxicity Test

Metabolic activity of viable cells was measured by MTT assay based on the reduction of 3-(4,5-dimethylthiazol-2-yl)-2,5-diphenyltetrazolium salt to a formazan product, which can be quantified spectrophotometrically to determine the percent of viable cells [32].

Anticancer activities of new benzothiazole acylhydrazones derivatives were evaluated against A549 C6, MCF-7 and HT-29 cell lines. The selectivity of their cytotoxic effects was evaluated on NIH/3T3 mouse embryonic fibroblast cells. NIH/3T3 cells were incubated in DMEM supplemented with fetal calf serum, penicillin (100 IU/mL), streptomycin (100 mg/mL) and 7.5% NaHCO_3_ at 37 °C in a humidified atmosphere of 95% air and 5% CO_2_. Carcinogenic cells were incubated in RPMI medium supplemented with fetal calf serum, penicillin (100 IU/mL), streptomycin (100 mg/mL) and 7.5% NaHCO_3_ at 37 °C in a humidified atmosphere of 95% air and 5% CO_2_. All cell lines were seeded at a density of 1 × 10^4^ cells into the 96-well plates. After 24 h of incubating period, the culture mediums were removed and test compounds were added at concentrations of 0.000316–1 mM. After a 24 h incubation period, OD of samples were measured by a microplate reader (Biotek, Winooski, VT, USA) at 540 nm. Inhibition % at concentrations was determined using the formula below and IC_50_ values were calculated by nonlinear regression analysis using the SigmaPlot v.10 package program (Manufacturer, City, US State abbrev. if applicable, Country) [33,34,35,36]. Cisplatin was used as a positive control:

% inhibition = 100 − (mean sample × 100/mean solvent)

#### 3.2.2. DNA Synthesis Inhibition Assay

The BrdU cell proliferation method was performed to analyse the effects of compounds **4d**, **4e** and **4h** on proliferation of C6 cells. C6 cells were seeded into the 96-well plates at a density of 1 × 10^4^ cells. Compounds were added into each well at five different concentrations (IC_50_/4, IC_50_/2, IC_50_, 2 × IC_50_, and 4 × IC_50_) and the plates were incubated for 24 h. At the end of the incubation period, BrdU solution was added and cells were reincubated for 2 h at 37 °C. Anti-BrdU-POD (100 mL) was added and the mixture was incubated for 90 min. Microplates were washed with PBS for three times. After adding substrate solution, the mixture was incubated for 15 min. OD of the wells were determined at 492 nm. Proliferation of control cells was assessed as 100% and growth inhibition % of cells, treated with test compounds and cisplatin were calculated [37].

#### 3.2.3. Flow Cytometric Analysis

Death pathway of the carcinogenic cell lines was detected by Annexin V-FITC Apoptosis Detection Kit (BD, Pharmingen, City, US State abbrev. if applicable, Country) as reported in the manufacturer’s instructions. Cisplatin and compounds **4d**, **4e**, and **4h**, which possess the highest cytotoxic activity, were used at their IC_50_/2, IC_50_, and 2 × IC_50_ concentrations. After 24 h incubation period, cells were harvested by centrifugation at 1200 rpm for 5 min. at room temperature, rinsed with cold water twice and then suspended at 1 × 10^6^ cells/mL concentration in Annexin V-FITC binding buffer. Propidium iodide (5 mL) and annexin V-FITC (5 mL) were added for staining the cells and the fluorescence measurements was performed using a flow cytometer. FCSExpress software (version, Manufacturer, City, US State abbrev. if applicable, Country) was used to display the percent of normal and apoptotic cells at different stages [37]. In the diagrams, Q1, Q2, Q3, and Q4 demonstrate the necrotic cells (positive for PI and negative for annexin/FITC), late apoptotic or necrotic cells (positive for annexin and PI), live cells (negative for annexin and PI), and apoptotic cells (negative for PI and positive for annexin), respectively. The experiments were carried out in triplicate.

## 4. Conclusions

A major challenge to medicinal chemists is determination of new structures that may be beneficial in the design of novel, selective and less-toxic anticancer agents. For this purpose, we synthesized new 2-((5-substituebenzothiazol-2-yl)thio)-*N*’-(2-(4-substitutedphenylethylidene)acetohydrazide (**4a**–**4j**) and evaluated their anticancer activity against A549, C6, MCF-7, and HT-29 cells. Cytotoxic activities of the synthesized compounds against healthy NIH3T3 cell lines were also investigated. It was determined that compounds **4d**, **4e**, and **4h** showed higher cytotoxicity than other compounds against C6 cell lines. Moreover, these compounds did not kill healthy cells at concentrations with cytotoxic effects against cancer cells, so it could be said that compounds **4d**, **4e**, and **4h** displayed selective inhibition towards cancer cells. Especially, the IC_50_ value of compound **4d** against NIH3T3 cell lines was calculated as 1 mM < similar to the IC_50_ value of cisplatin. Structures of the test compounds vary from each other in terms of substituents on BT and phenyl substructures. Evaluating the substituent effect on cytotoxic activity, compounds **4a**–**4e** bearing 5-chlorobenzothiazole have been noted with greater effects. In addition, 4-(4-methylpiperidin-1-yl)phenyl moiety in compound **4d** caused a selective cytotoxic effect.

Consequently, anticancer activity screening of novel benzothiazole-acylhydrazones indicated that compound **4d**, which is the lead compound of the series, possesses significant selective cytotoxic effects on cancer cells. Furthermore, compound **4d** shows higher antiproliferative activity than cisplatin. However, it has induced apoptosis in cancer cells, dose-dependently. On the other hand, compound **4e** (SI = 0.33), which has lower selectivity index (SI) compared to **4d**, induced apoptotic cell death in cancer cells. At this point, we could be emphasized that findings of this preliminary study will not only direct us to synthesize similar compounds with greater anticancer activity, but also may have an influence on medicinal chemists to synthesize more active compounds.

## Figures and Tables

**Table:** 

Compounds	R_1_	R_2_	R_3_	R_4_	X
**4a**	-H	-H	-H	-Cl	-CH
**4b**	-CH_3_	-H	-H	-Cl	-CH
**4c**	-H	-CH_3_	-H	-Cl	-CH
**4d**	-H	-H	-CH_3_	-Cl	-CH
**4e**	-H	-H	4-methoxyphenyl	-Cl	-N
**4f**	-H	-H	-H	-OCH_3_	-CH
**4g**	-CH_3_	-H	-H	-OCH_3_	-CH
**4h**	-H	-CH_3_	-H	-OCH_3_	-CH
**4i**	-H	-H	-CH_3_	-OCH_3_	-CH
**4j**	-H	-H	4-methoxyphenyl	-OCH_3_	-N

**Table 1:** Cytotoxic activity of the compounds against A549, C6, MCF-7, HT-29 and NIH3T3 cell lines.

Compounds	IC_50_ (mM)				
	A549	C6	MCF-7	HT-29	NIH3T3
**4a**	1<	0.10	0.49	0.52	0.10
**4b**	1	0.10	1<	1	0.10
**4c**	0.52	0.10	0.10	0.30	0.03
**4d**	1<	0.03	0.10	0.30	1<
**4e**	0.03	0.03	0.30	1<	0.01
**4f**	1<	1<	1<	1<	0.03
**4g**	1<	1<	1<	1<	0.10
**4h**	1<	0.03	1<	1<	0.10
**4i**	0.49	0.10	0.30	1	0.03
**4j**	1<	1<	1<	1<	1<
**Cisplatin**	0.06	0.03	0.05	0.06	1<

**Table 2:** The flow cytometric analysis results of compounds **4d**, **4e**, and **4h** and cisplatin for C6 cell line.

Compound	IC_50_/2				IC_50_				2 × IC_50_			
	Q1	Q2	Q3	Q4	Q1	Q2	Q3	Q4	Q1	Q2	Q3	Q4
**4d**	16.4	12.3	71.2	0.1	6.9	3.1	87.6	2.4	4.5	7.1	74.5	13.9
**4e**	13.5	3.8	82.6	0	9.8	16.0	70.7	3.5	10.1	14.0	73.3	2.5
**4h**	10.8	4.9	84.1	0.3	8.6	7.4	79.4	4.6	5.4	10.5	75.2	8.9
**Cisplatin**	N.D.	N.D.	N.D.	N.D.	3.5	18.3	75.6	2.6	N.D.	N.D.	N.D.	N.D.

N.D.: Not Determined.

## Supplementary Materials

The following are available online, Spectra 1-12: IR, HRMS, ^1^H-NMR, and ^13^C-NMR spectra of compounds **4d**, **4e** and **4h**.
