# Supplementary material for: Synthesis of New Benzothiazole Acylhydrazones as Anticancer Agents
Source: Molecules. 2018 May 1;23(5):1054. doi: 10.3390/molecules23051054 (PMC6102592; doi:10.3390/molecules23051054)
Supplement: Supplementary file 1 [file molecules-23-01054-s001.zip › molecules-289912-supplementary.pdf]

DOPNALAB

	Item	Value
	Acquired Date&Time	23.12.2016 15:53:23
	Acquired by	System Administrator
	Filename	C:\Users\dopnalab\Desktop\derya\ZD serisi\ZD-61.ispd
	Spectrum name	ZD-61
	Sample name	ZD-6
	Sample ID	
	Option	
	Comment	
	No. of Scans	10
	Resolution	4 [cm-1]
	Apodization	Happ-Genzel

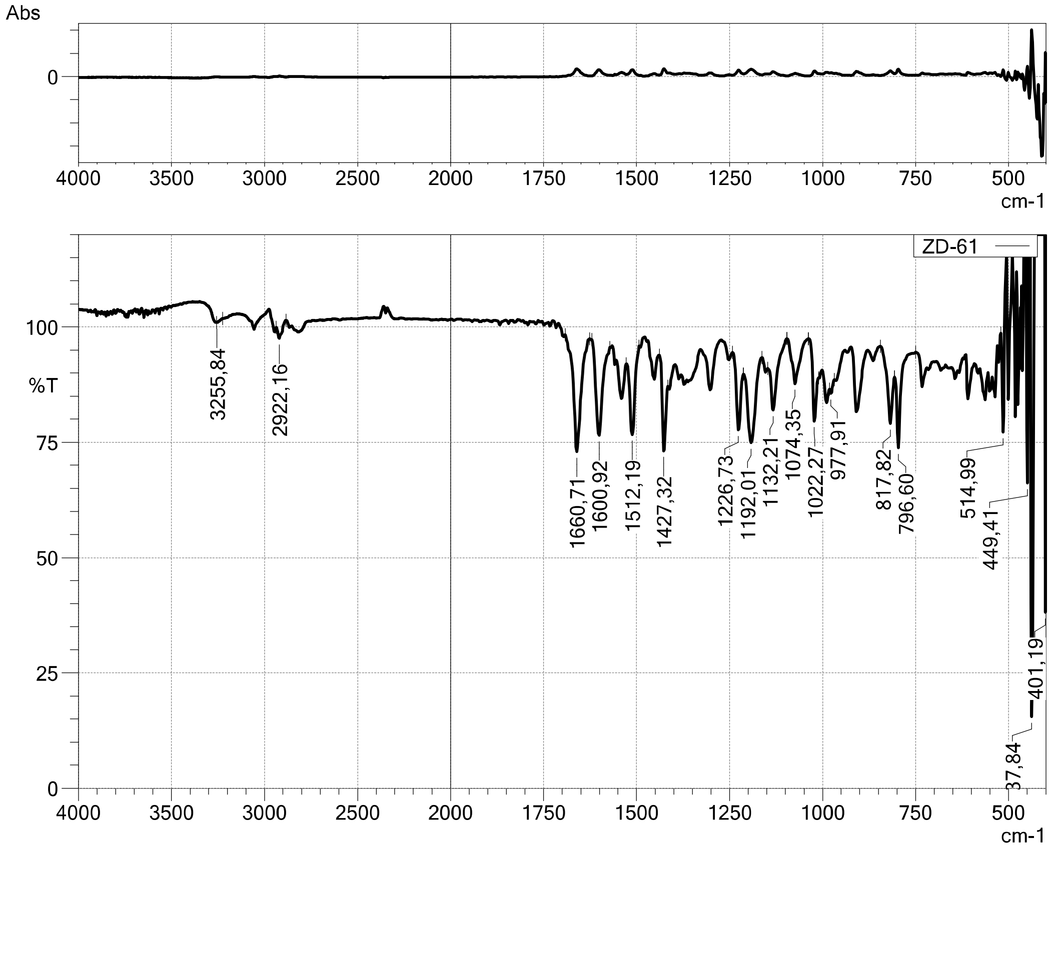

Spectra 1. Compound **4d** IR spectra.

Data File: C:\ LabSolutions\ Data\Analiz\derya\ ZD-6_33.lcd

Elmt	Val.	Min	Max	Elmt	Val.	Min	Max	Elmt	Val.	Min	Max	Elmt	Val.	Min	Max	Use Adduct
H	1	4	30	O	2	1	3	S	2	0	3	Ru	2	0	0	H
C	4	5	33	F	1	0	0	Cl	1	0	1	I	3	0	0	
N	3	2	5	P	3	0	0	Br	1	0	0					

Error Margin (ppm):	15	DBE Range:	10.0 - 15.0	Electron Ions:	both
HC Ratio:	unlimited	Apply N Rule:	yes	Use MSn Info:	no
Max Isotopes:	3	Isotope RI (%):	1.00	Isotope Res:	10000
MSn Iso RI (%):	10.00	MSn Logic Mode:	AND	Max Results:	500

Event#: 1 MS(E+) Ret. Time : 9.160 -> 9.160 Scan# : 1375 -> 1375

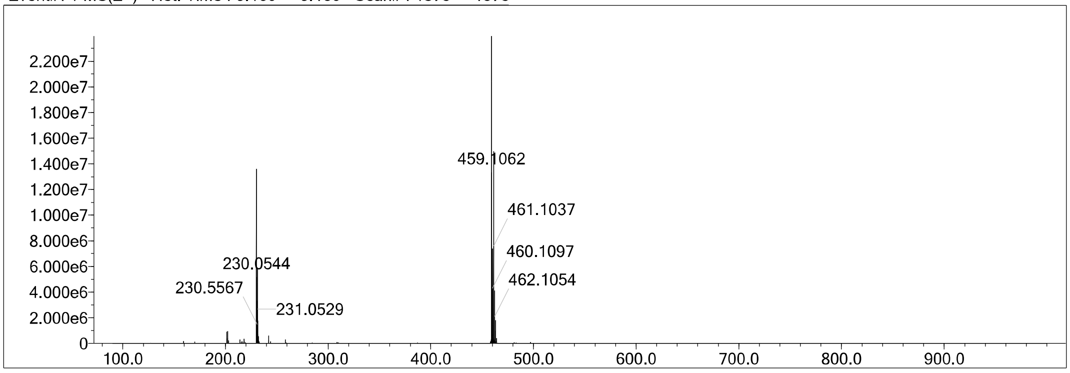

Measured region for 459.1062 m/z

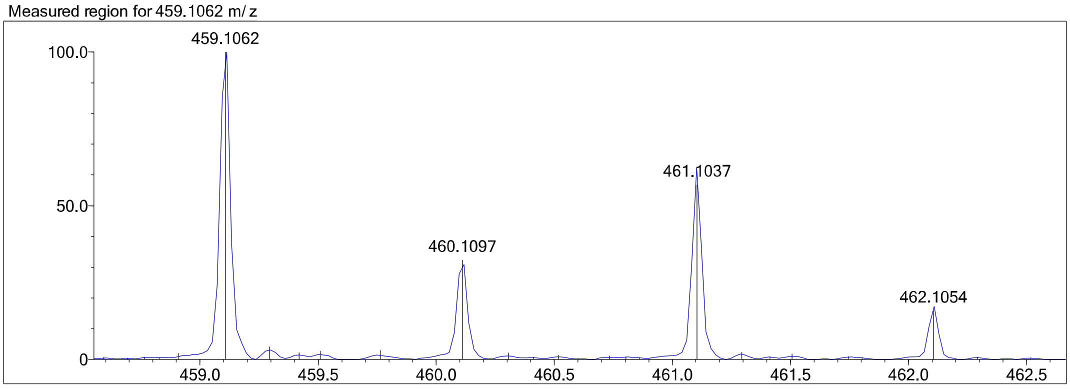

C22 H23 N4 O S2 Cl [M+H]+ : Predicted region for 459.1075 m/z

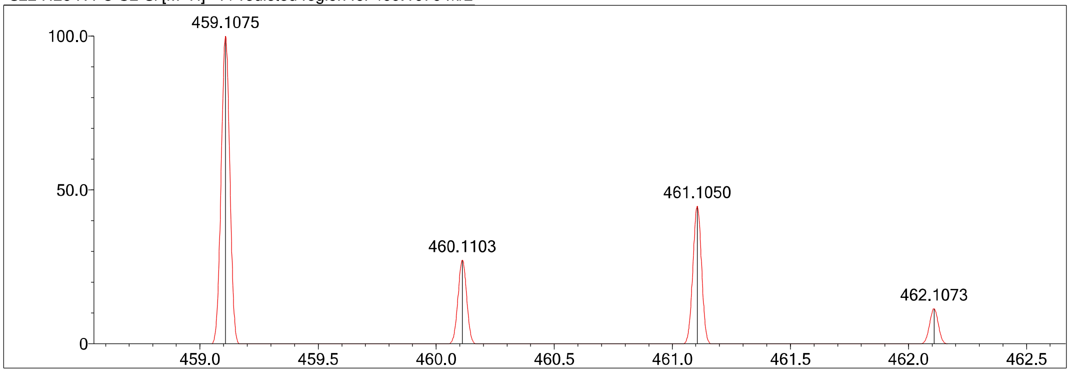

Rank	Score	Formula (M)	Ion	Meas. m/z	Pred. m/z	Df. (mDa)	Df. (ppm)	Iso	DBE
1	64.71	C22 H23 N4 O S2 Cl	[M+H]+	459.1062	459.1075	-1.3	-2.83	67.81	13.0

Spectra 2. Compound **4d** HRMS spectra.

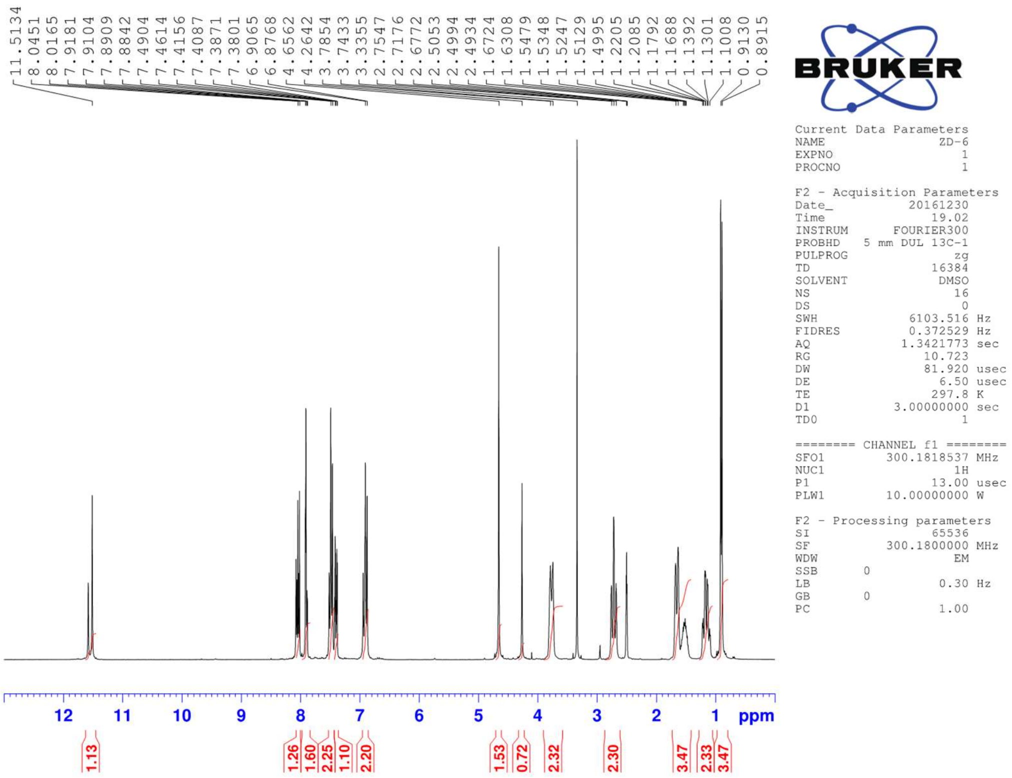

Spectra 3. Compound **4d** ^{1}H-NMR spectra.

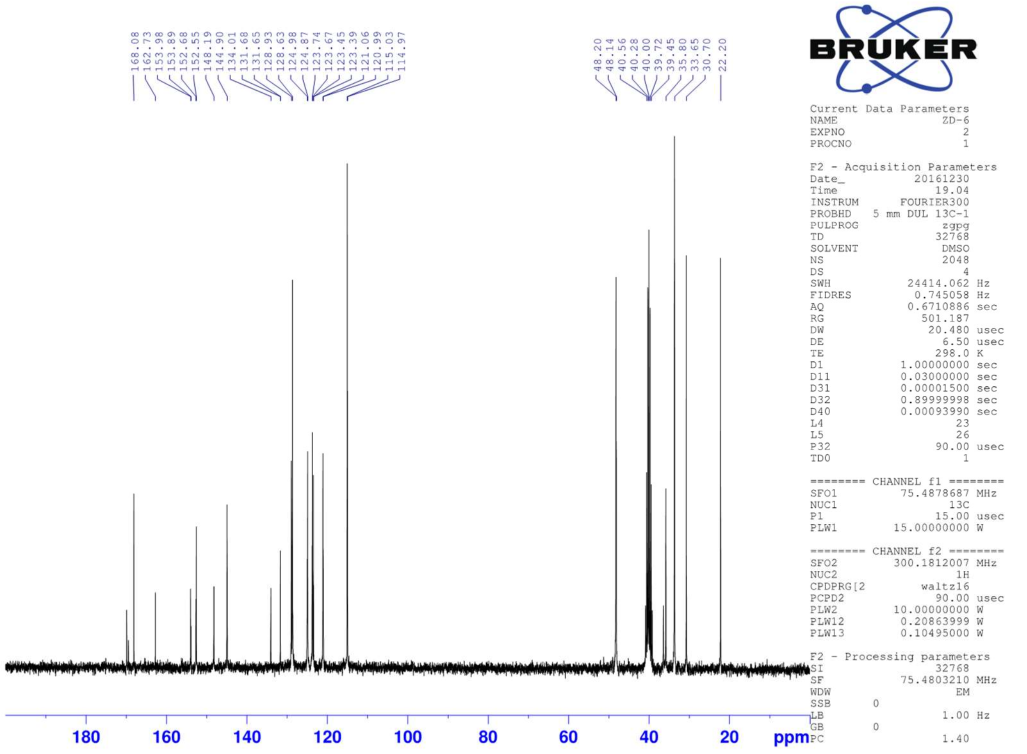

Spectra 4. Compound **4d** ^{13}C-NMR spectra.

DOPNALAB

	Item	Value
	Acquired Date&Time	23.12.2016 15:03:05
	Acquired by	System Administrator
	Filename	C:\Users\dopnalab\Desktop\derya\ZD serisi\ZD-21.ispd
	Spectrum name	ZD-21
	Sample name	ZD-2
	Sample ID	
	Option	
	Comment	
	No. of Scans	10
	Resolution	4 [cm-1]
	Apodization	Happ-Genzel

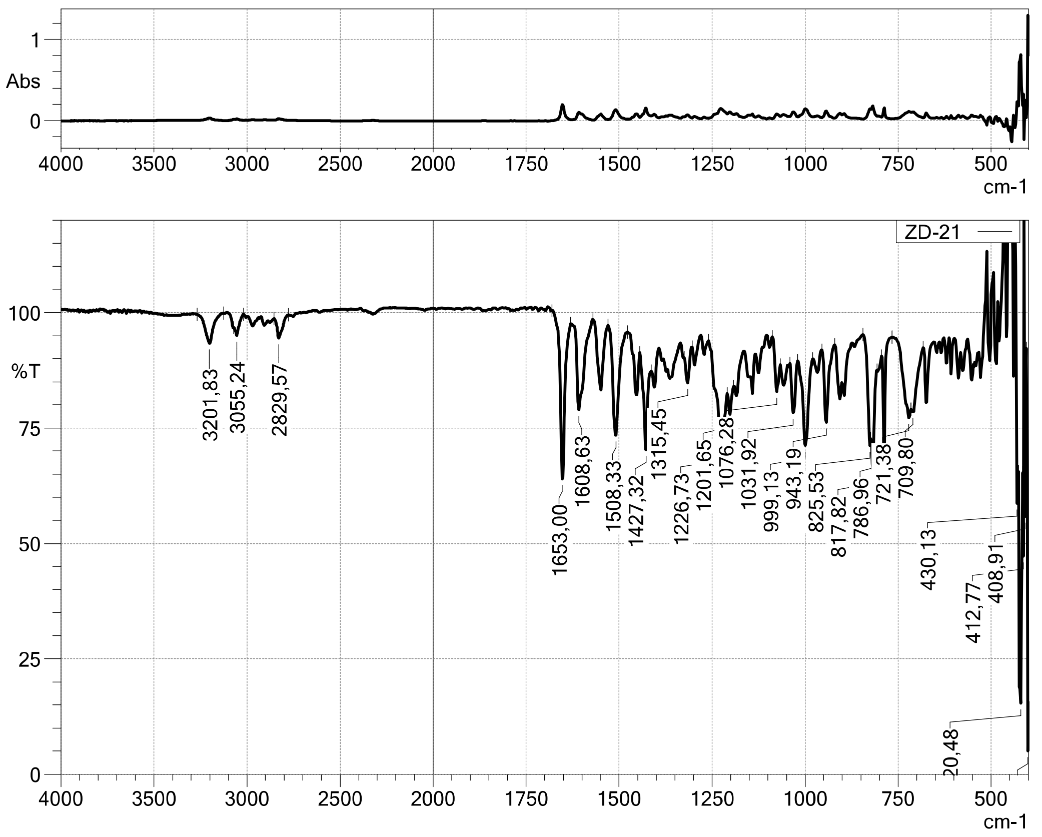

Spectra 5. Compound **4e** IR spectra.

Data File: C:\ LabSolutions\ Data\Analiz\derya\ ZD-2_29.lcd

Elmt	Val.	Min	Max	Elmt	Val.	Min	Max	Elmt	Val.	Min	Max	Elmt	Val.	Min	Max	Use Adduct
H	1	4	30	O	2	1	3	S	2	0	3	Ru	2	0	0	H
C	4	5	33	F	1	0	0	Cl	1	0	1	I	3	0	0	
N	3	2	6	P	3	0	0	Br	1	0	0					

Error Margin (ppm):	15	DBE Range:	15.0 - 18.0	Electron Ions:	both
HC Ratio:	unlimited	Apply N Rule:	yes	Use MSn Info:	no
Max Isotopes:	3	Isotope RI (%):	1.00	Isotope Res:	10000
MSn Iso RI (%):	10.00	MSn Logic Mode:	AND	Max Results:	500

Event#: 1 MS(E+) Ret. Time : 8.320 -> 8.320 Scan# : 1249 -> 1249

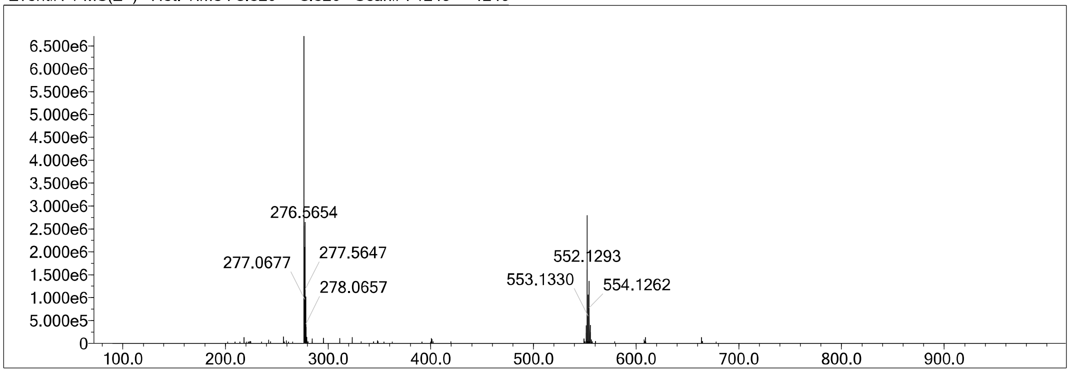

Measured region for 552.1293 m/z

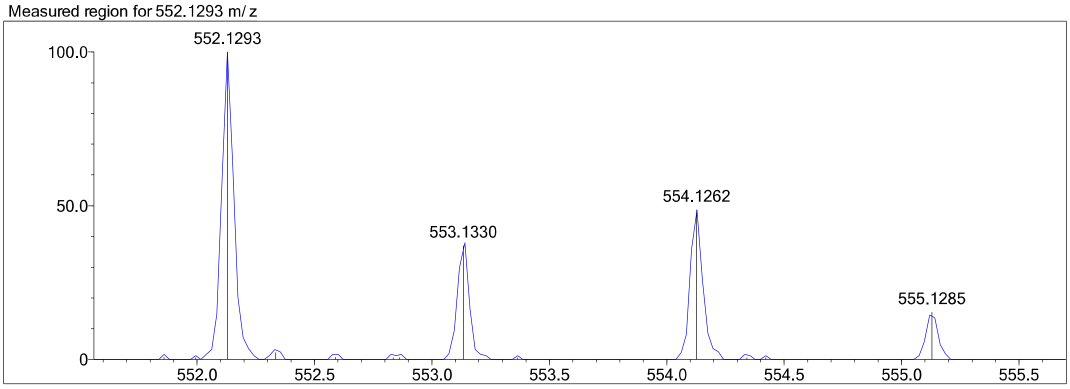

C27 H26 N5 O2 S2 Cl [M+H]+ : Predicted region for 552.1289 m/z

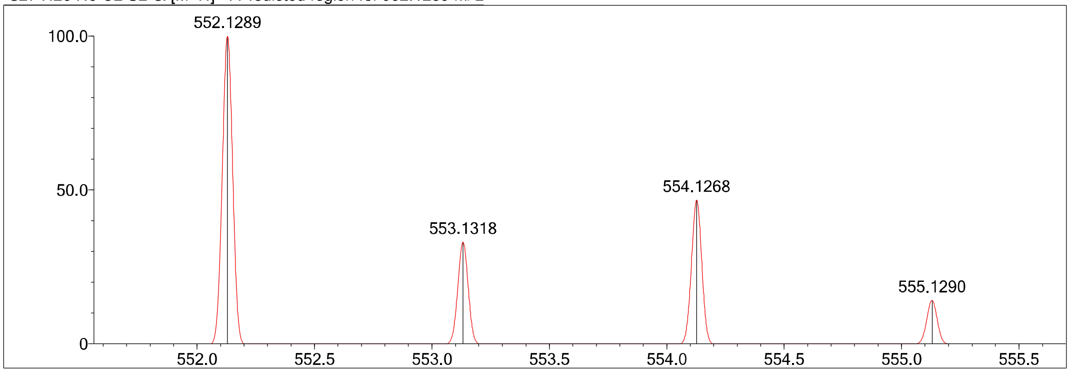

Rank	Score	Formula (M)	Ion	Meas. m/z	Pred. m/z	Df. (mDa)	Df. (ppm)	Iso	DBE
1	100.00	C27 H26 N5 O2 S2 Cl	[M+H]+	552.1293	552.1289	0.4	0.72	100.00	17.0

Spectra 6. Compound **4e** HRMS spectra.

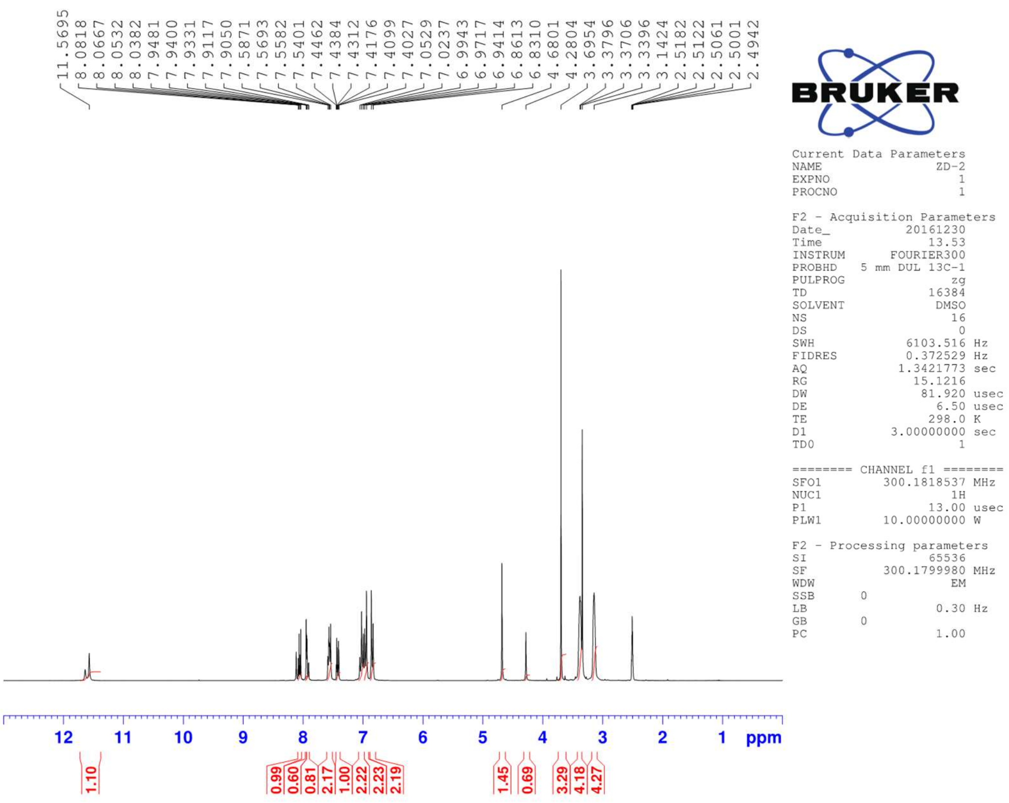

Spectra 7. Compound **4e** ^{1}H-NMR spectra.

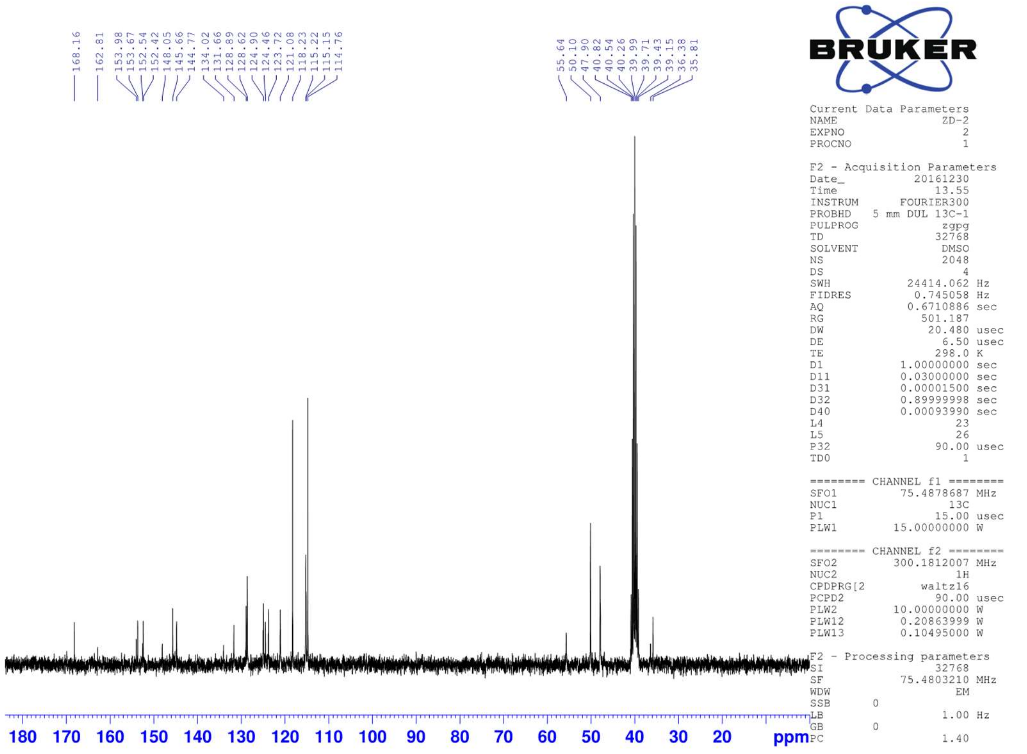

Spectra 8. Compound **4e** ^{13}C-NMR spectra.

DOPNALAB

	Item	Value
	Acquired Date&Time	23.12.2016 16:04:09
	Acquired by	System Administrator
	Filename	C:\Users\dopnalab\Desktop\derya\ZD serisi\ZD-111.ispd
	Spectrum name	ZD-111
	Sample name	ZD-11
	Sample ID	
	Option	
	Comment	
	No. of Scans	10
	Resolution	4 [cm-1]
	Apodization	Happ-Genzel

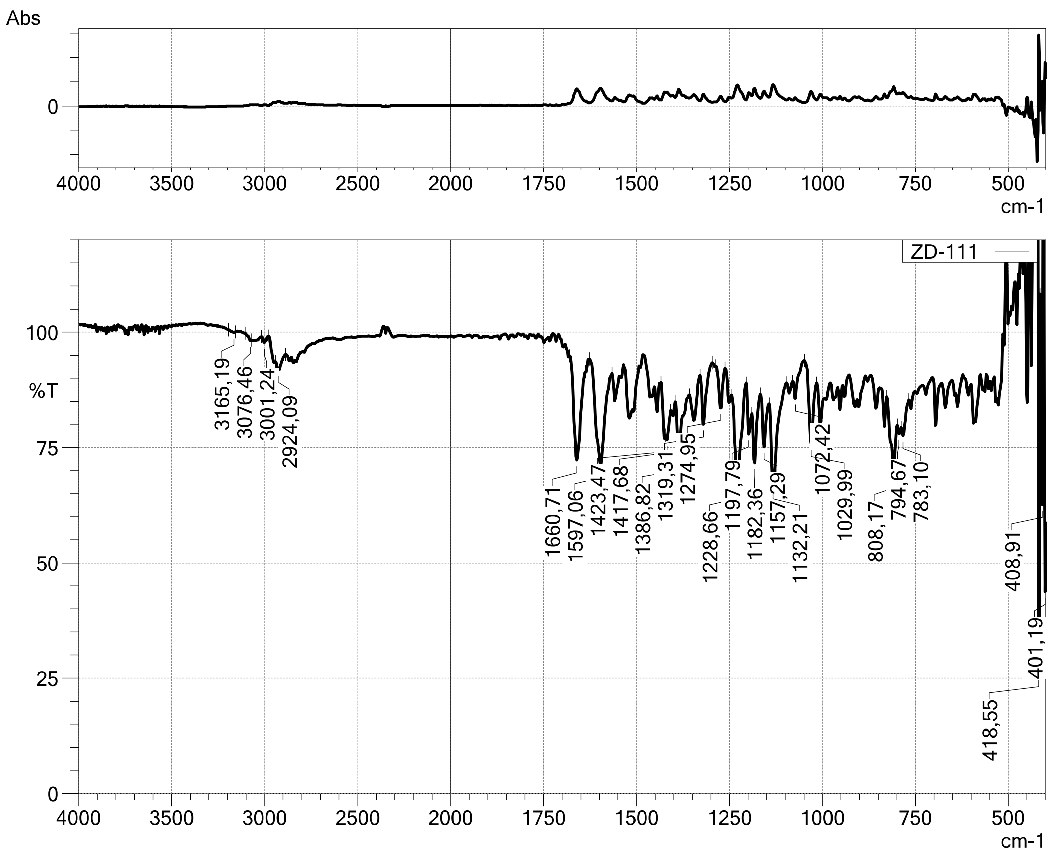

Spectra 9. Compound **4h** IR spectra.

Data File: C:\ LabSolutions\ Data\Analiz\derya\ ZD-11_38.lcd

Elmt	Val.	Min	Max	Elmt	Val.	Min	Max	Elmt	Val.	Min	Max	Elmt	Val.	Min	Max	Use Adduct
H	1	4	30	O	2	1	3	S	2	0	3	Ru	2	0	0	H
C	4	5	33	F	1	0	0	Cl	1	0	0	I	3	0	0	
N	3	2	5	P	3	0	0	Br	1	0	0					

Error Margin (ppm):	10	DBE Range:	10.0 - 15.0	Electron Ions:	both
HC Ratio:	unlimited	Apply N Rule:	yes	Use MSn Info:	no
Max Isotopes:	3	Isotope RI (%):	1.00	Isotope Res:	10000
MSn Iso RI (%):	10.00	MSn Logic Mode:	AND	Max Results:	500

Event#: 1 MS(E+) Ret. Time : 8.627 Scan# : 1295

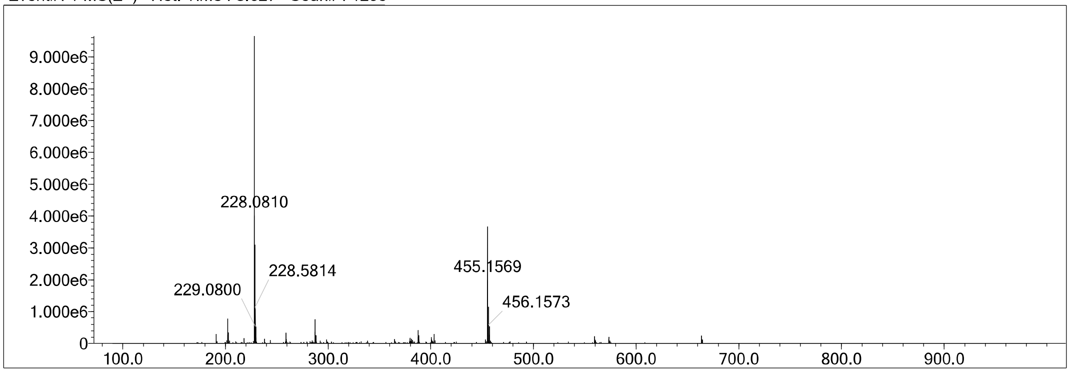

Measured region for 455.1569 m/z

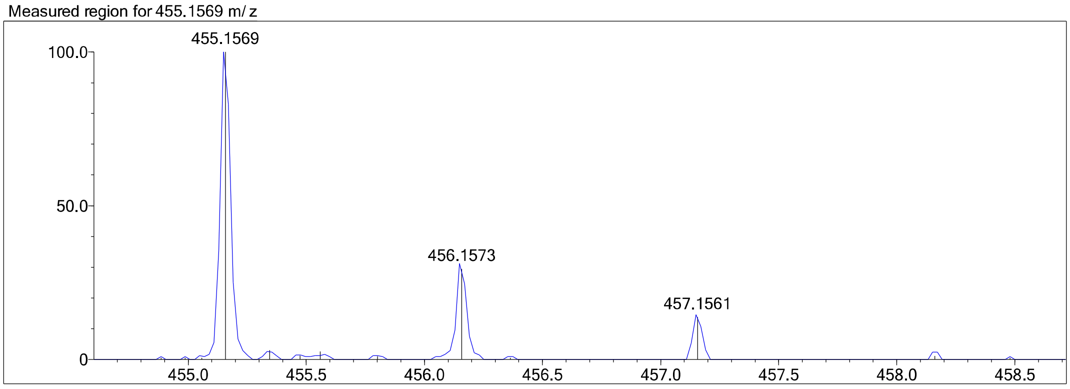

C23 H26 N4 O2 S2 [M+H]+ : Predicted region for 455.1570 m/z

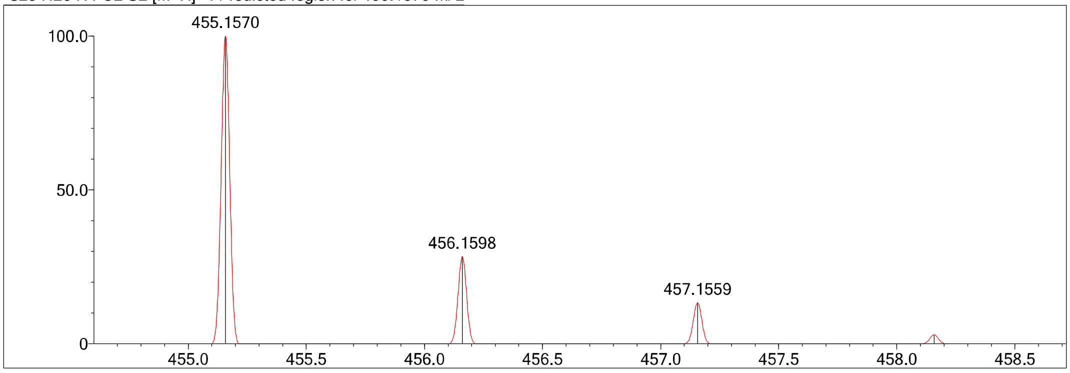

Rank	Score	Formula (M)	Ion	Meas. m/z	Pred. m/z	Df. (mDa)	Df. (ppm)	Iso	DBE
1	83.68	C23 H26 N4 O2 S2	[M+H]+	455.1569	455.1570	-0.1	-0.22	83.68	13.0

Spectra 10. Compound **4h** HRMS spectra.

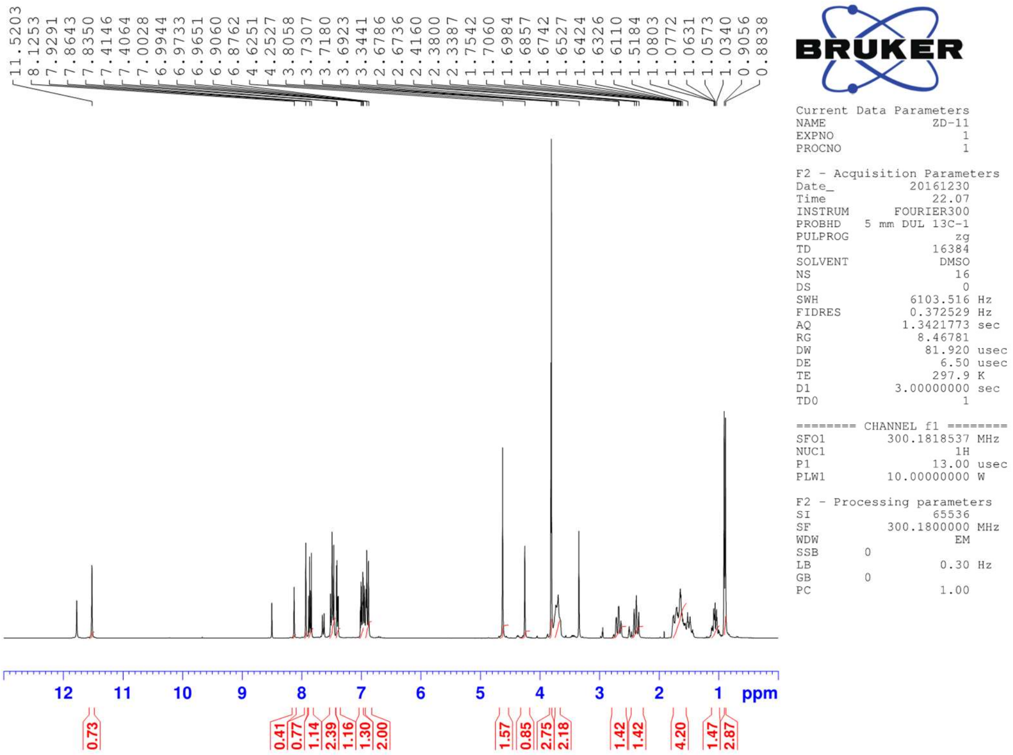

Spectra 11. Compound **4h** ^{1}H-NMR spectra.

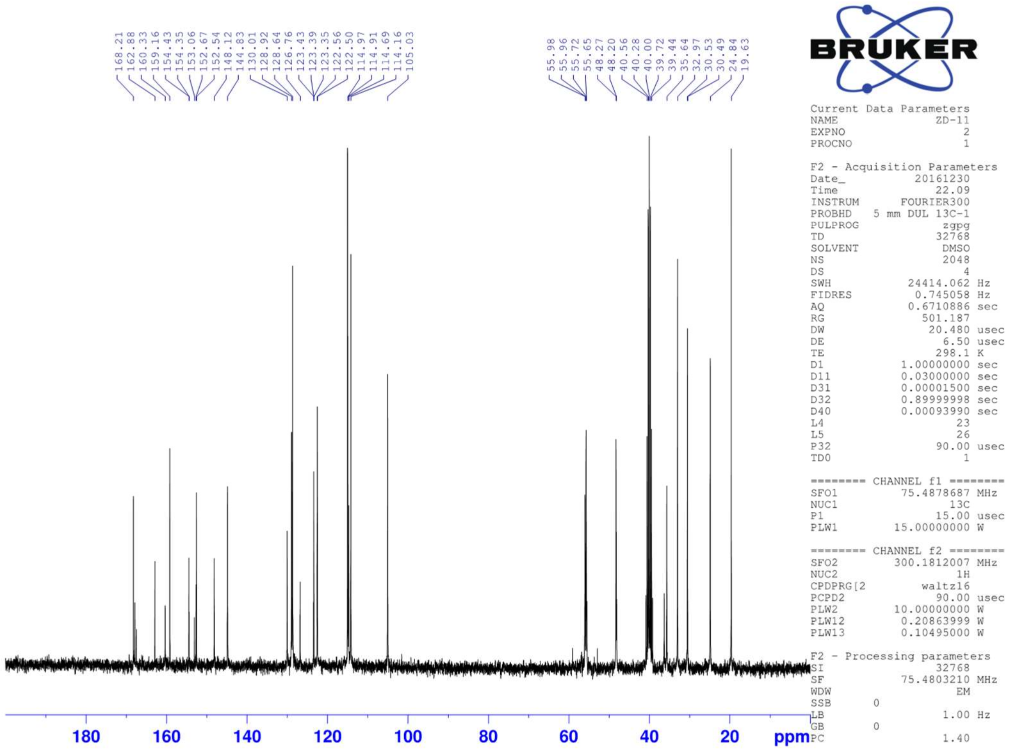

Spectra 12. Compound **4h** ^{13}C-NMR spectra.
